# Common patterns between dengue cases, climate, and local environmental variables in Costa Rica: A wavelet approach

**DOI:** 10.1371/journal.pgph.0002417

**Published:** 2023-10-19

**Authors:** Yury E. García, Shu Wei Chou-Chen, Luis A. Barboza, Maria L. Daza–Torres, J. Cricelio Montesinos-López, Paola Vásquez, Juan G. Calvo, Miriam Nuño, Fabio Sanchez

**Affiliations:** 1 Department of Public Health Sciences, University of California Davis, CA, United States of America; 2 Centro de Investigación en Matemática Pura y Aplicada - Escuela de Estadística, Universidad de Costa Rica, San José, Costa Rica; 3 Centro de Investigación en Matemática Pura y Aplicada, Universidad de Costa Rica, San José, Costa Rica; 4 Centro de Investigación en Matemática Pura y Aplicada - Escuela de Matemática, Universidad de Costa Rica, San José, Costa Rica; Portland State University, UNITED STATES

## Abstract

Dengue transmission poses significant challenges for public health authorities worldwide due to its susceptibility to various factors, including environmental and climate variability, affecting its incidence and geographic spread. This study focuses on Costa Rica, a country characterized by diverse microclimates nearby, where dengue has been endemic since its introduction in 1993. Using wavelet coherence and clustering analysis, we performed a time-series analysis to uncover the intricate connections between climate, local environmental factors, and dengue occurrences. The findings indicate that multiannual dengue frequency (3 yr) is correlated with the Oceanic Niño Index and the Tropical North Atlantic Index. This association is particularly prominent in cantons located along the North and South Pacific Coast, as well as in the Central cantons of the country. Furthermore, the time series of these climate indices exhibit a leading phase of approximately nine months ahead of dengue cases. Additionally, the clustering analysis uncovers non-contiguous groups of cantons that exhibit similar correlation patterns, irrespective of their proximity or adjacency. This highlights the significance of climate factors in influencing dengue dynamics across diverse regions, regardless of spatial closeness or distance between them. On the other hand, the annual dengue frequency was correlated with local environmental indices. A persistent correlation between dengue cases and local environmental variables is observed over time in the North Pacific and the Central Region of the country’s Northwest, with environmental factors leading by less than three months. These findings contribute to understanding dengue transmission’s spatial and temporal dynamics in Costa Rica, highlighting the importance of climate and local environmental factors in dengue surveillance and control efforts.

## 1 Introduction

Dengue is a mosquito-borne viral infection caused by four antigenically distinct dengue virus serotypes (DENV1–4). The transmission to humans occurs by the bite of an infected female mosquito. Functioning as the primary vector, *Aedes aegypti* is extensively spread across tropical and sub-tropical regions globally and is well adapted to thrive in urban environments. *Aedes albopictus*, identified as one of the 100 most harmful invasive species worldwide [1], assumes a secondary vector role and is present on all continents except Antarctica [2, 3].

Dengue is a flu-like illness that affects individuals of all ages, causing significant health, economic, and social burdens on a population [4]. The clinical profile of patients can range from asymptomatic infection to severe cases.

In recent years, the complex interaction of biological, socioeconomic, environmental, and climatic factors has facilitated the rapid emergence of dengue worldwide, becoming endemic and a relevant public health problem in more than 100 countries [5]. In the last decades, the number of dengue cases reported to the World Health Organization has increased from 505,430 in 2000 to more than 4.2 million in 2019 [5, 6].

Seasonal case patterns and vector abundance suggest that dengue transmission is sensitive to climatic and environmental factors [7, 8]. Climatic conditions can alter spatial and temporal dynamics of vector ecology, potentially increasing vector ranges, lengthening the duration of vector activity, and increasing the mosquito’s infectious period [7]. Precipitation provides habitats for the aquatic stages of the mosquito life cycle and strongly influences vector abundance and distribution [8, 9]. Water temperature plays a significant role in mosquito reproduction since it directly affects survival at all stages of its life cycle [10]. Laboratory studies have shown that optimal temperature for survival of *Ae. aegypti* in all life stages ranges from 20° to 30°C. At a temperature of 25°C, the virus replication, maturation, and migration process to the salivary glands, enabling mosquito-to-human transmission, typically takes between 5 and 33 days (average 15 days). Temperature increments are associated with higher vector viral replication rates and shorter extrinsic incubation periods [7]. The time lag between changes in precipitation, temperature, and subsequent effects on dengue outbreaks vary from 7 to 15 weeks [7]. Higher humidity increases *Ae. aegypti* feeding activity and enhances disease spread.

Dengue’s complex transmission dynamics have motivated the study of the correlation of cases with meteorological and ecological variables [11–13]. Most of these works evaluated the effects and correlation between dengue cases and climate variables [14, 15] and seasonal vegetation dynamics, which may also influence the biology of the vector populations at relatively local scales [16, 17]. Barrera et al. [18] suggested that dense vegetation can promote *Ae. aegypti* pupal productivity by contributing organic material to the habitat, influencing water temperature and evaporation by creating shades.

This study focuses on Costa Rica, a Central American country renowned for its diverse microclimates. Since 1993, the dengue virus has been observed within the country, with coastal regions particularly susceptible to its high prevalence. Over the years, there have been significant fluctuations in transmission peaks across affected regions, posing substantial challenges to public health efforts to develop effective mitigation strategies.

The primary objective of this study is to explore the correlation between dengue cases and a range of climatic and local environmental factors in 32 specific cantons, which are of particular interest to the Minister of Health in Costa Rica. To achieve this, we use wavelet coherence and wavelet cluster analysis techniques. The examined factors include El Niño Southern Oscillation, Tropical North Atlantic Index, Normalized Difference Water Index, Enhanced Vegetation Index, Evapotranspiration, and precipitation. The wavelet transform method enables the decomposition of time series data into distinct components within the time and frequency domains. This approach facilitates the identification of variations occurring across different periods, offering valuable insights into non-stationary signals. Furthermore, this method highlights the synchronicity of series during specific periods.

The utilization of wavelets has gained significant popularity in analyzing time series data, with numerous applications across various domains. These applications encompass the exploration of characteristics within non-stationary time series [12, 13, 19], examination of spatial patterns [11, 13], the investigation of associations between population and environmental time series, identification of phase and synchrony patterns [12, 13], as well as the analysis of multiple time series [20, 21].

## 2 Materials and methods

### 2.1 Study area

**Fig 1:**
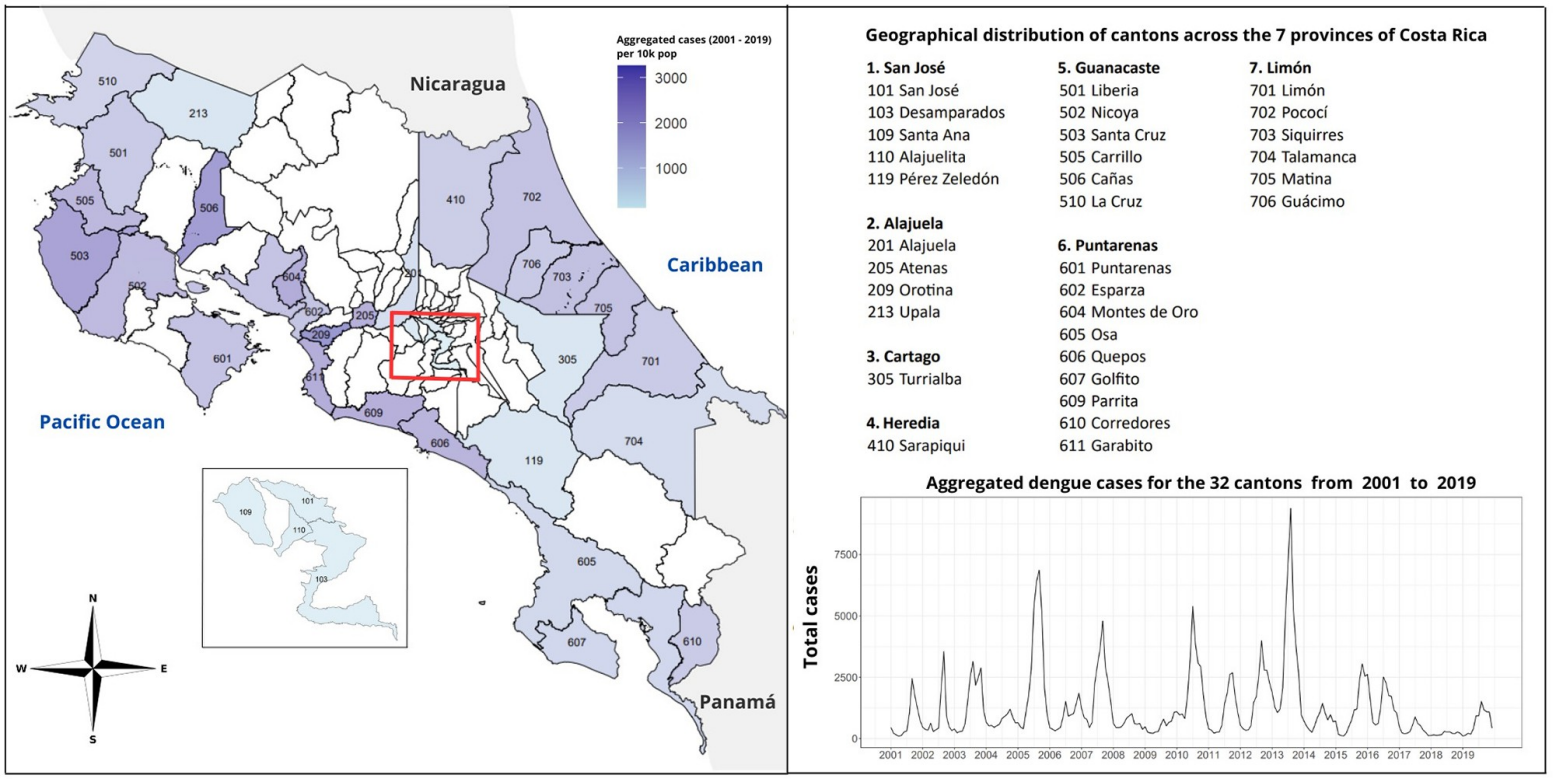
Map of aggregated dengue cases per 10,000 population in Costa Rica’s 32 cantons (left panel). Time series of aggregated dengue cases from 32 Cantons (7 Provinces, right-panel). (Shapefiles are publicly available at [60]).

In Central America, Costa Rica shares borders with Nicaragua to the North, the Caribbean Sea to the Northeast, Panama to the Southeast, and the Pacific Ocean to the Southwest. Costa Rica spans a land area of 51,060 square kilometers and has a population of approximately five million people. The population is densely aggregated in the Great Metropolitan Area, including the capital San José. Costa Rica is administratively divided into seven provinces: San José, Alajuela, Heredia, Cartago, Guanacaste, Puntarenas, and Limón. These provinces are further divided into different Cantons (83 in total). For our analysis, we focused on 32 cantons (colored in Fig 1) identified by local public health authorities as areas of interest, considering the significant number of dengue cases reported.

The country exhibits a tropical climate characterized by a remarkable diversity of microclimates. Along the Pacific Coast and Central Region, a distinct dry season prevails from December to April, followed by a rainy season extending from May to November, with abundant rainfall. Conversely, the climate in the Eastern plains and coasts and the Southern part of the Pacific coast can be described as equatorial, with substantial rainfall occurring throughout the year.

In the 1950s, Costa Rica successfully combatted the *Ae. aegypti* mosquito, leading to the country being declared free of the vector by 1961. However, influenced by the lack of consistent active surveillance, positive locations for the presence of the mosquito were again detected around 1971. Consequently, a new eradication campaign was launched. Unfortunately, by 1992, the vector had already spread to nearly all regions of the country. In September 1993, the first dengue cases were reported on the Pacific Coast, marking the onset of endo-epidemic transmission with the circulation of all four dengue virus serotypes [22].

According to data from the Ministry of Health, between 1993 and 2021, a total of 398,546 dengue cases have been reported in Costa Rica. Over these years, transmission has exhibited both seasonal and inter-annual variability, with the highest number of cases reported in 2013 (49,993 cases), followed by 2005 (37,798 cases) and 2010 (31,484 cases) [23]. While the coastal regions have been the most affected areas, trends observed over the years indicate variations in transmission peaks across all affected regions. Detailed information on the distribution of cases over time and geography can be found in the supplementary material (Figs A-C in S1 Text).

### 2.2 Data

#### 2.2.1 Dengue cases

Data from 2001 to 2019, containing weekly dengue case records for all cantons, was made available by the Ministry of Health in Costa Rica. To better capture the annual cycles present in the data, we aggregated the information to a monthly level for this study. This approach was preferred over using weekly data, as weekly data could introduce more measurement errors and increase variability, potentially leading to confusing or misleading patterns. Data was also square-root transformed, and standardized for this analysis due to their asymmetry [13].

#### 2.2.2 Climate variables

The Tropical North Atlantic (TNA) Index [24] and El Niño Southern Oscillation (ENSO) Index for region 3.4 [25] were utilized to evaluate anomalies in the Caribbean and the Pacific Ocean, respectively. The TNA Index provides information on sea surface temperatures in the eastern tropical North Caribbean Ocean. On the other hand, El Niño, the positive phase of the ENSO, refers to a climate phenomenon characterized by the warming of sea surface temperatures in the central and eastern tropical Pacific Ocean. It is often associated with significant global weather pattern changes, including shifts in precipitation patterns and the occurrence of extreme events such as droughts or heavy rainfall [26]. El Niño generally takes place approximately every 3–7 years and persists for an average period of 9–12 months [27]. Based on the ENSO Index, El Niño events are identified when the index values surpass one, whereas La Niña events occur when the values drop below one. El Niño has well-documented effects on Costa Rica, resulting in decreased rainfall on the Pacific slope and increased precipitation patterns on the Atlantic slope. Henceforth, we will employ the terms TNA and El Niño 3.4 as climate variables rather than referring to them as the TNA Index and El Niño 3.4 Index. TNA and El Niño 3.4 will be recognized and discussed as climate variables.

#### 2.2.3 Environmental variables

The Normalized Difference Water Index (NDWI) is used to assess the presence and abundance of water content in vegetation and land surfaces. Elevated values of the NDWI index suggest a greater presence of water bodies or moisture content [28]. The Enhanced Vegetation Index (EVI) is commonly used to assess the health and vigor of vegetation cover on Earth’s surface. Higher values of EVI indicate an increased extent of healthy and green vegetation in the study area [29]. Evapotranspiration (ET) Index is a measurement used to assess the efficiency of water use by plants and vegetation. It indicates the amount of water evaporated from the soil and transpired by plants relative to the amount of water available for evapotranspiration. Higher values of ET reflect a higher rate of water loss, indicating more active evaporation and transpiration processes in the region [30]. Finally, the CHIRPS precipitation index gives the estimated amount of rainfall in millimeters for each grid cell over a specific period, usually monthly. These grid cells cover the Earth’s surface, and by aggregating the data, we can obtain insights into the distribution and intensity of rainfall in different regions [31].

All data were standardized to make them comparable to the results of other time series. An average of each index was calculated and mapped using data from 2001 to 2019; Maps illustrate local environmental changes across the country and are presented in the supplementary material (Fig P in S1 Text). Going forward, we will utilize EVI, NDWI, ET, and Precipitation as the terms for our discussions, omitting the word “Index”. These four variables will be recognized as local environmental variables or factors, and it should be noted that each canton has its unique time series data for these variables.

### 2.3 Wavelets analysis

Wavelet analysis is well-suited for examining noisy and non-stationary data, making it suitable for studying dengue case data characterized by pronounced seasonality and interannual variability (yearly changes) [32, 33]. In the following sections, we present a brief introduction to wavelet theory. However, for a comprehensive understanding of wavelet analysis techniques, we refer to the study conducted by Torrence and Compo [34], which offers an extensive presentation. Additionally, previous studies have explored the application of these techniques in ecological and epidemic scenarios, as described by Cazelles et al. [33, 35, 36].

#### 2.3.1 Wavelet power spectra

Wavelet analysis is based on the transform:

$$\begin{array}{c} W_{x}\left(s,\tau \right)=\frac{1}{\sqrt{s}}\int_{-\infty }^{\infty }x\left(t\right)\Psi^{*}(\frac{t-\tau }{s})dt=\int_{-\infty }^{\infty }x\left(t\right)\Psi_{s,\tau }^{*}\left(t\right)dt, \end{array}$$ (1)

where * denotes the complex conjugate form and Ψ_*s*,*τ*_(*t*) represent a family of functions derived from a single function called the “mother wavelet”. The signal is decomposed in these functions, which can be expressed in terms of two parameters, one for the time position *τ*, and the other for the scale of the wavelets *s*, given by

$$\begin{array}{c} \Psi_{s,\tau }\left(t\right)=\frac{1}{\sqrt{s}}\Psi (\frac{t-\tau }{s}). \end{array}$$ (2)

For this analysis, we use the R-packages *WaveletComp* [37] that analyze the frequency structure of uni- and bivariate time series using the Morlet mother wavelet [33, 35]

$$\begin{array}{c} \Psi \left(t\right)=\pi^{\frac{-1}{4}}e^{i\omega t}e^{\frac{-t^{2}}{2}}. \end{array}$$ (3)

This leads to a continuous complex-valued wavelet transform that can be separated into its real and imaginary parts, providing information on both local amplitude and instantaneous phase of any periodic process across time, a prerequisite for investigating multiple time series coherence [37].

#### 2.3.2 Analyzing two time series

Cross-wavelet and wavelet coherence allowed us to compare two-time series, such as climate and dengue, and to identify synchronous frequencies or signals. The cross-wavelet transform of two-time series *x*(*t*) and *y*(*t*), with respective wavelet transforms *W*_*x*_ and *W*_*y*_, decomposes the Fourier co- and quadrature-spectra in the time-frequency (or time-scale) domain

$$W_{x,y}\left(\tau ,s\right)=\frac{1}{s}W_{x}\left(\tau ,s\right)W_{y}^{*}\left(\tau ,s\right).$$

The concepts of cross-wavelet analysis provide a tool for (i) comparing the frequency of two-time series, (ii) concluding the series’ synchronicity at specific frequencies and across certain ranges of time [37]. Its modulus can be interpreted as cross-wavelet power.

$$Power.xy\left(\tau ,s\right)=|W_{x,y}\left(\tau ,s\right)|.$$

In a geometric sense, the cross-wavelet transform is the analog of the covariance. However, it lends itself to certain limitations for interpretation concerning the degree of association of the two series that can be remedied by coherence.

#### 2.3.3 Wavelet coherence

Wavelet coherence is a statistical tool that identifies synchronization patterns or correlations between two-time series that are not easily observable through traditional time-domain analysis. It achieves this by computing the wavelet transform of each time series, which converts them into a series of coefficients representing the signal’s intensity at different frequencies and time scales. These coefficients are then used to measure the strength of the correlation between the two-time series at each frequency and time scale, resulting in the wavelet coherence measure.

In contrast to the cross-wavelet transform, wavelet coherence is a normalized measure of dependence that can construct confidence intervals, which are often easier to interpret [37]. It is analogous to the Fourier coherency measure, which determines the cross-correlation between two-time series as a function of frequency. Wavelet coherence is the equivalent concept in wavelet theory, similar to the geometric sense of coherence in classical correlation. Wavelet coherence is calculated as the squared coherence and is analogous to the coefficient of determination in statistics.

$$\begin{array}{c} \;R_{x,y}\left(\tau ,s\right)=\frac{|<W_{x,y}{\left(\tau ,s\right)>|}^{2}}{|<W_{x}{\left(\tau ,s\right)>|}^{2}{\left|<W_{y}\left(\tau ,s\right)>\right|}^{2}}. \end{array}$$ (4)

The angle brackets indicate smoothing in both time and frequency, *W*_*x*_(*τ*, *s*) and *W*_*y*_(*τ*, *s*) are the wavelet transform of the series *x*(*t*) and *y*(*t*), respectively, and *W*_*x*,*y*_(*τ*, *s*) is the cross-wavelet transform. The value of *R*_*x*,*y*_(*τ*, *s*) range between 0 and 1, where 1 represent a perfect linear relationship between the time series *x*(*t*) and *y*(*t*).

The two signals’ phase differences provide information about series synchronization (i.e., in or out of phase). The Morlet wavelet is complex so that the phase difference can be computed in terms of the real (R) and the imaginary (I) part, as

$$\begin{array}{c} \Phi_{x,y}\left(\tau ,s\right)=\frac{I(<W_{x,y}\left(\tau ,s\right)>)}{R(<W_{x,y}\left(\tau ,s\right)>)}. \end{array}$$ (5)

The phase difference of two-time series *x*(*t*) and *y*(*t*) can provide valuable information about the degree of synchronization between the two series. By computing the phase difference at each localizing time origin and scale, we can determine whether the two series are in-phase or out-of-phase, representing an angle in the [−*π*, *π*] range.

#### 2.3.4 Wavelet clusters

Clustering involves dividing a set of objects into groups or clusters based on their similarity, where objects within the same cluster are more similar than objects in other clusters. Traditional clustering algorithms often rely on certain assumptions to define subgroups within a dataset. Consequently, evaluating the validity of the resulting clustering scheme becomes necessary.

Given this study’s time-dependency and non-stationarity of the dengue and climatic data, we recognized the need for a specialized clustering analysis approach. To address these challenges, we utilized a methodology based on vector wavelet coherence, introduced by Oygur and Unal [38]. This approach provides a means to assess the synchronicity and co-movements between various climatic time series and dengue data. It enables us to perform cluster analysis based explicitly on their synchronicity patterns across the 32 cantons.

To perform the cluster analysis based on vector wavelet coherence, we utilized Wald’s method [39]. By constructing a dissimilarity matrix using vector wavelet coherence, we were able to compute various clustering indices, including Silhouette [40], Frey [41], McClain [42], Cindex [43], and Dunn [44]. We considered a range of 2 to 15 clusters to determine the optimal number of clusters. However, it is important to acknowledge that these clustering indices may not always agree on the optimal number of clusters. We took a combined approach in light of the potential discrepancies among the clustering indices. We utilized a quantitative metric, the Cindex criterion, alongside our practical understanding to decide the optimal number of clusters.

#### 2.3.5 Computing environment

All statistical analyses were conducted using R version 2.4 (www.R-project.org) [45]. The wavelet coherence analysis was performed by utilizing the *vwc* function from the R-package vectorwavelet [38]. Dissimilarity matrices were computed using the *wclust* function in the R-package biwavelet [46], which enabled us to measure the dissimilarity between wavelet coherence results. The creation of clusters and clustering indices were calculated using the NbClust package [47]. To obtain more interpretable results regarding the correlation between dengue incidence and each climatic and local environmental variable, we utilized the WaveletComp package *version 1.1* [37]. This package facilitated the visualization and grouping of wavelet coherence, power average, and phase difference based on the clusters obtained using the biowavelet package.

#### 2.3.6 Results presentation and interpretation

The wavelet coherence is represented by a colored plot. These figures describe the degree of correlation and synchronization of two time series at different frequencies (y-axis) and time scales (x-axis). Areas of high coherence are usually displayed in warm colors (e.g., red or yellow), while areas of low coherence are shown in cool colors (e.g., blue or green). The solid white outline indicates the 95% confidence levels of statistically significant areas, where the null hypothesis of “no periodicity” is rejected at a default significance level of 10% (*p*<0.1). The cones of influence, displayed as lighter-shaded regions, represent the areas where edge effects can increase the uncertainty of the analysis.

Wavelet coherence figures were created by dividing power levels into quintiles to achieve an even color distribution, but this method may sometimes exaggerate artifacts in the data. An additional image is generated within the white outlines to accurately represent the significant area and prevent artifacts in the image plot. This image shows the phase differences between the two-time series. Arrows indicate synchronization, both in and out of phase, providing insights into the temporal relationship between the variables. Right-pointing horizontal arrows indicate that the two series are in phase at the respective frequency with vanishing phase differences, while left-pointing arrows indicate they are out of phase. The angle of the arrow can be used to identify the leading time series: angles in [0, *π*/2] or in [−*π*/2, −*π*] indicate that the leading time series is dengue, whereas angles in [0, −*π*/2] or in [*π*/2, *π*] indicate that climatic or environmental variables lead. This approach was described in Rösch et al. [37]. In the context of wavelet coherence analysis, when we observe that a climatic or local environmental variable precedes the dengue time series, it indicates that variations or changes in the climatic variable occur before changes in the number of dengue cases.

## 3 Results

### 3.1 Wavelet coherence of dengue cases and climate variables

**Fig 2:**
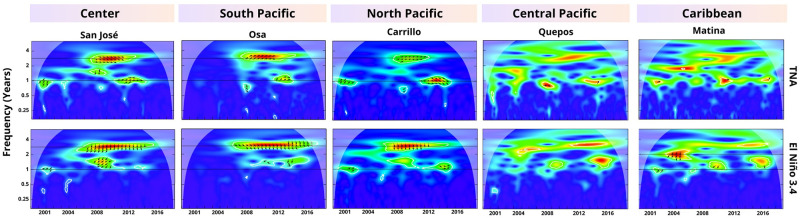
Wavelet coherence of dengue and climate variables. The figures illustrate the correlation (area scaled from yellow to red within white outline) between TNA and El Niño 3.4 with dengue cases at frequencies of three and one year. The arrows indicate the series that leads in each time interval.

**Fig 3:**
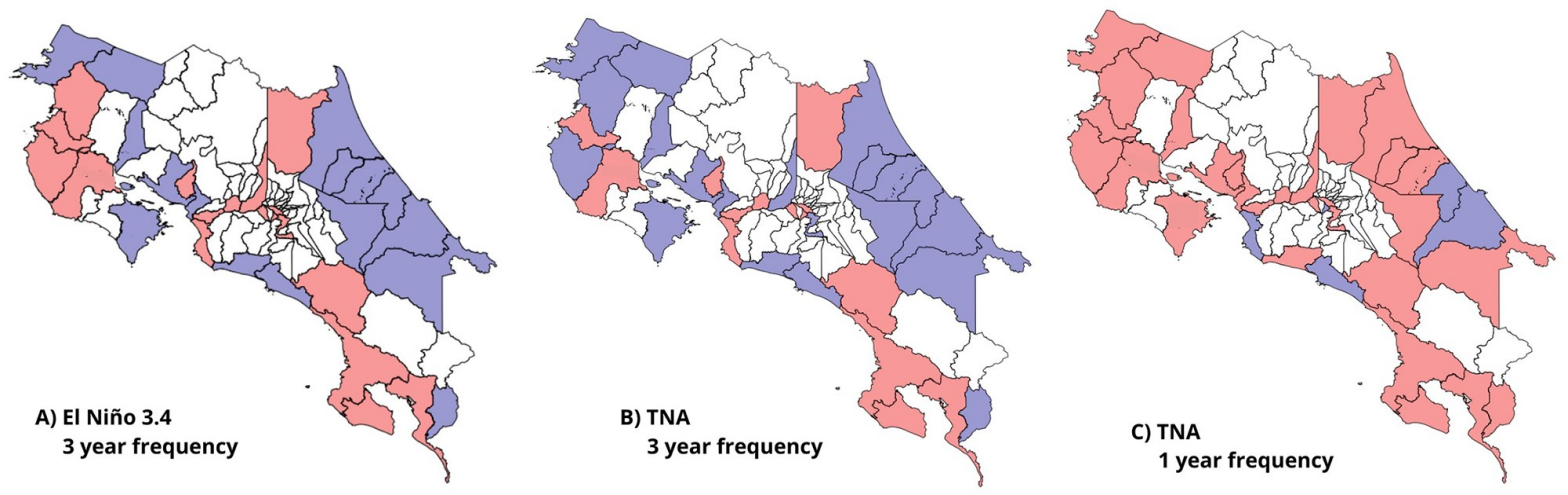
Significant correlation between El Niño, TNA, and dengue cases. Fig A and Fig B depict cantons (highlighted in red) that exhibit a significant correlation between El Niño, TNA, and dengue cases at a 3-yr frequency. Similarly, Fig C displays the cantons with a correlation between TNA and dengue cases at a frequency of 1 year. (Shapefiles are publicly available at [60]).

Fig 2 illustrates wavelet coherence of dengue cases and climate variables in cantons located in different country’s regions (wavelet coherence plots remaining cantons are presented in the Figs D-H in S1 Text). The relationship between the climatic factors, TNA and El Niño 3.4, and dengue cases exhibit variations both in space and time. Despite this variability, certain patterns consistently emerge in regions where a correlation has been established: 1) Multiannual dengue frequency, particularly at 3-yr, exhibits correlations with anomalies in Pacific and Caribbean regions. This correlation is particularly prominent in cantons located in the central region of the country, as well as those close to the North and South Pacific Ocean (Fig 3, Figs D-H in S1 Text). Besides, this correlation is not evident in cantons primarily in the Caribbean region and others such as Parrita and Quepos in the central Pacific. The time series demonstrate synchronized movement, with the climate time series leading by approximately nine months. 2) Correlation between climate and dengue cases is also observed at the 1, 1.5, and 2-yr frequencies, with the dengue time series leading. 3) Except for Quepos, Garabito, Limón, and Matina (Fig 3), the annual dengue frequency shows a correlation with TNA. In these cases, the time series move in phase, but there are brief periods where the leading or lagging time series change relative to each other. Notice that the correlation is not stationary.

### 3.2 Wavelet coherence of dengue cases and environmental variables

**Fig 4:**
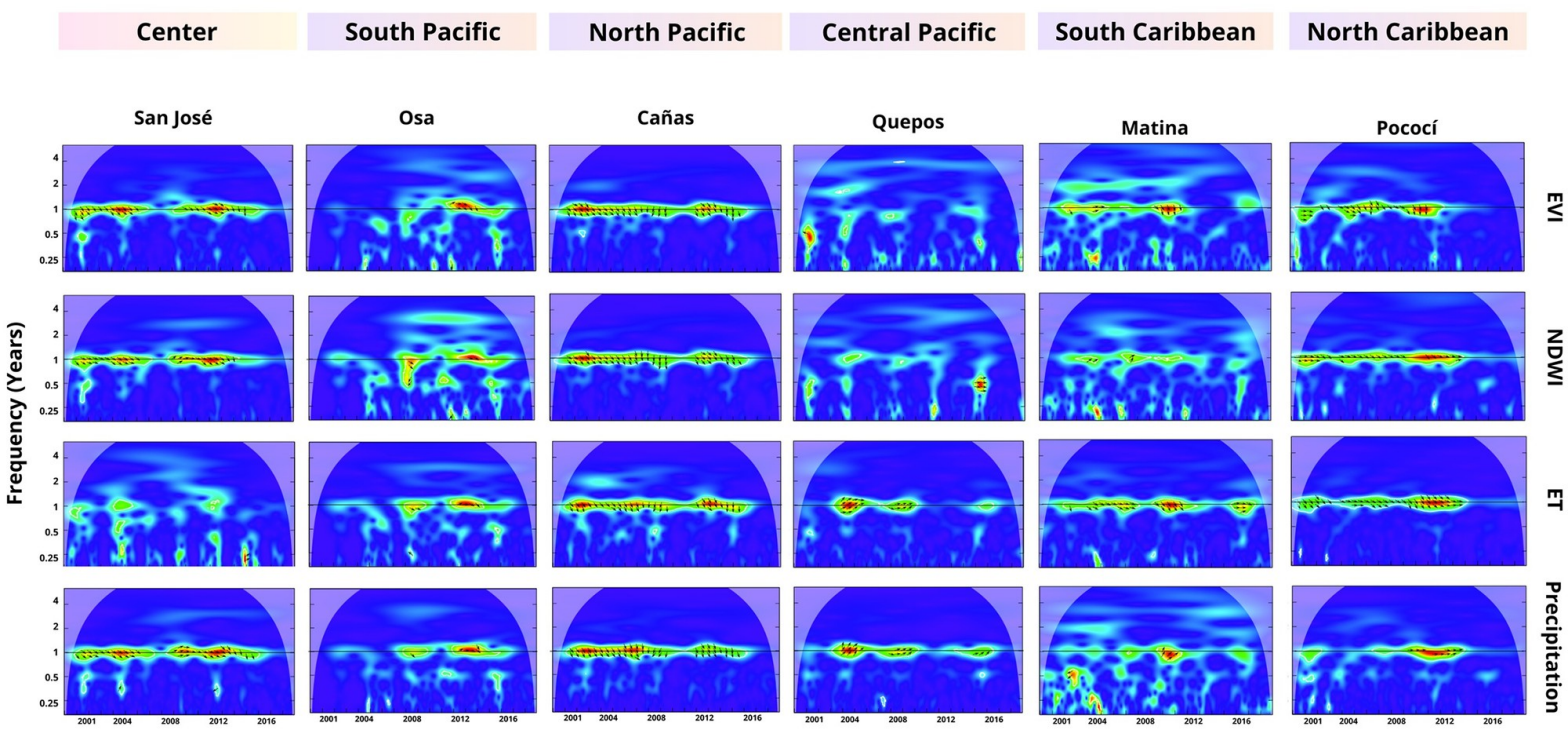
Wavelet coherence of dengue and environmental variables. The figures illustrate the annual correlation (area scaled from yellow to red within white outline) between EVI, NDWI, ET, and Precipitation with dengue cases. The arrows indicate the series that leads in each time interval.

**Fig 5:**
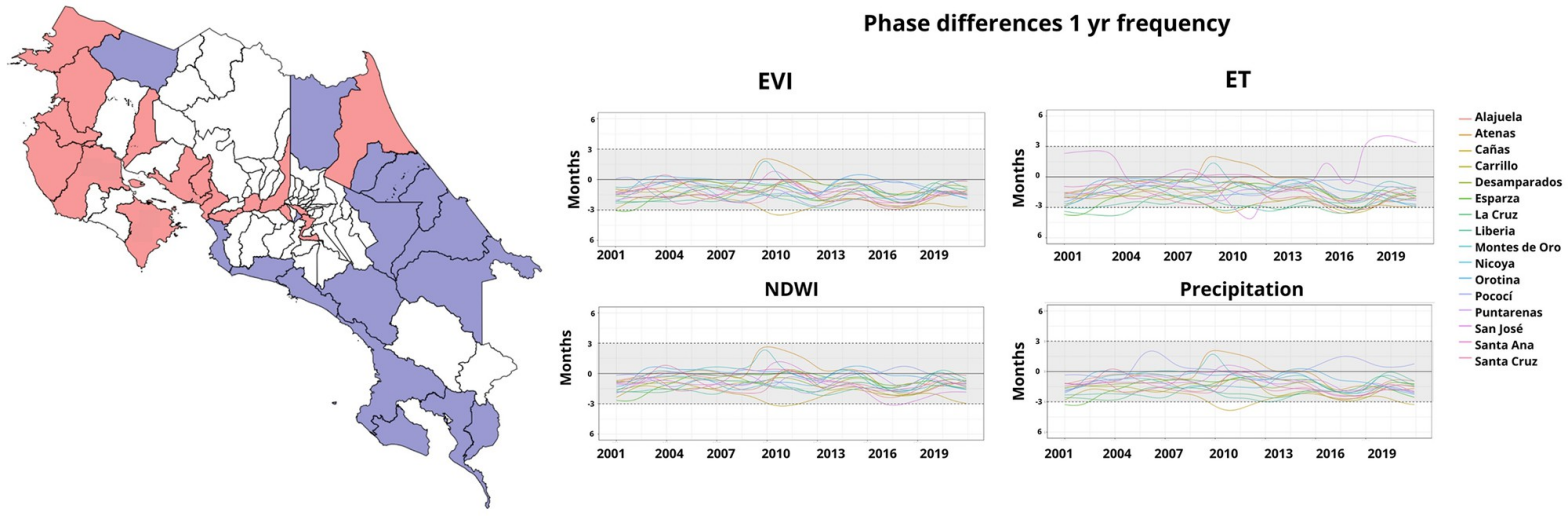
Significant correlation and temporal synchronization of dengue cases and environmental variables. Map on the left side highlights cantons (in red) where the annual correlation between dengue cases and local environmental variables was observed over time. Panels on the right side illustrate the phase difference between the dengue time series and each of the local environmental variables, revealing patterns of synchronization and time lag. (Shapefiles are publicly available at [60]).

The wavelet coherence analysis revealed a significant statistical correlation between the annual dengue frequency and the local environmental variables. The time series displayed synchronized movement patterns, with the leading and lagging time series changing over time and across cantons (Fig 4 and Figs I-M in S1 Text). The analysis also shows correlation over multiple years (see Fig 4 for a visual representation of the identified areas of high significance in San José and Cañas) in cantons predominantly located in the North Pacific and the central part of the country’s Northwest region, (Fig 5). Specifically in Alajuela, Montes de Oro, Orotina, Pococí, Santa Ana, Cañas, Puntarenas, Carrillo, La Cruz, Santa Cruz, Esparza, Desamparados, San José, Liberia, Nicoya, and Atenas. In these specific cantons, the time series consistently exhibited synchronized behavior with the environmental time series leading by less than three months (Fig 5).

In contrast, we did not observe any evident relationship between the EVI and dengue cases in the cantons of Parrita, Limón, Sarapiquí, Talamanca, Golfito, Garabito, Quepos, and Upala. Similarly, no significant correlation was found between the NDWI and dengue cases in the cantons of Matina, Talamanca, Garabito, Quepos, Parrita, and Corredores. Additionally, the ET did not correlate with dengue cases in the cantons of San José and Parrita. Furthermore, precipitation did not significantly correlate with dengue cases in Limón, Matina, Osa, Siquirres, and Talamanca (these cantons are blue-colored in Fig O in S1 Text).

### 3.3 Cluster analysis

We employed wavelet coherence-based clustering analysis to identify patterns and relationships between time series data in different regions. Our results reveal that cantons grouped into clusters share similar frequency patterns and onsent of time series synchronization. The initial analysis incorporated all variables, encompassing climatic and local environmental factors. However, the resulting outcomes were not easily interpretable, with most clusters comprising a single canton. Considering these findings, we conducted separate analyses for climatic and local environmental variables. It was observed that climatic variables displayed significant correlations with dengue cases at a frequency of 3 years, while local environmental variables exhibited notable correlations at a frequency of 1 year. This approach allowed us to derive clearer and more meaningful interpretations of the results.

**Fig 6:**
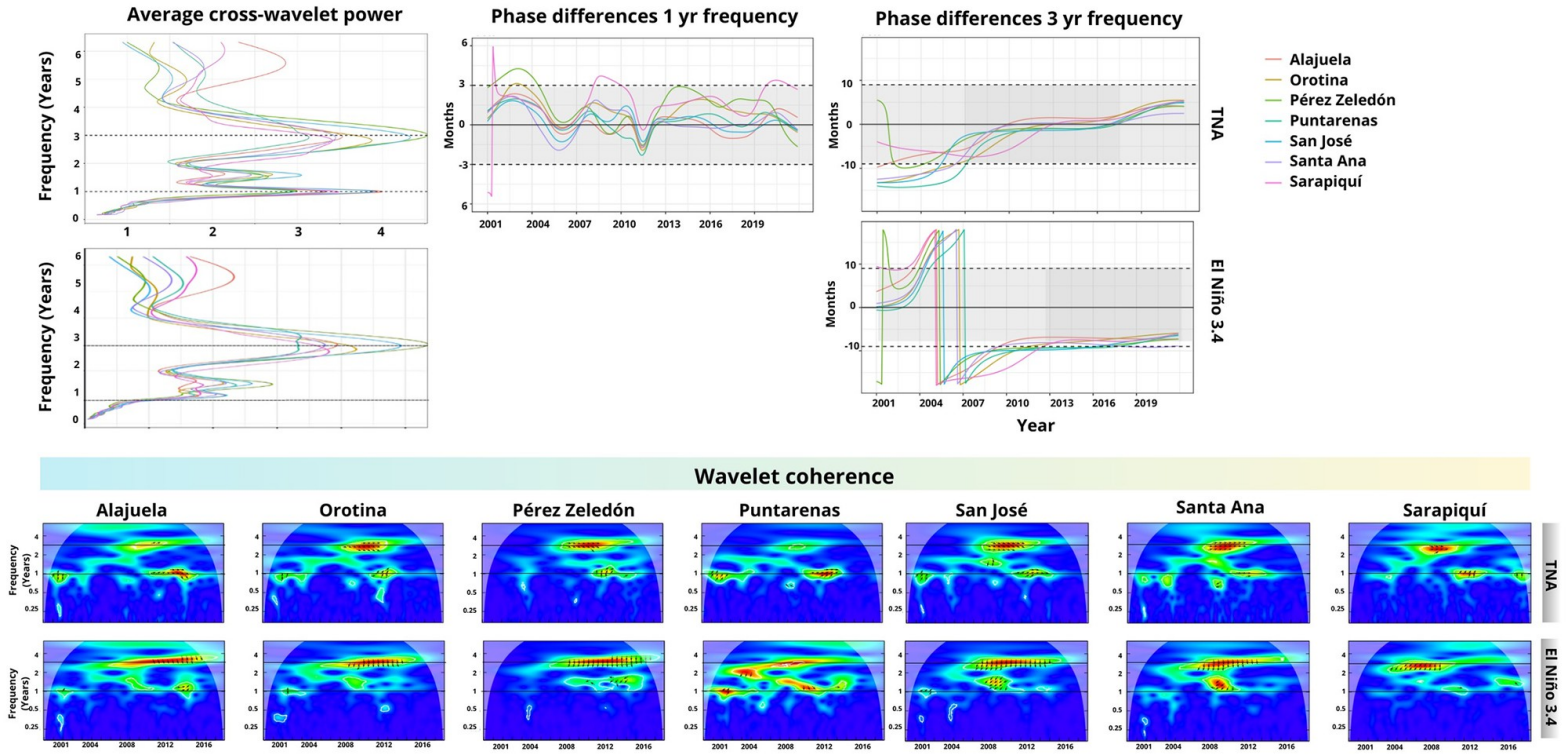
Wavelet coherence results cluster 4. The average cross-wavelet power (top-left) highlights the synchronized frequency in years, further illustrated by the coherence of the wavelets that also display the synchronization time points between the signals. The phase difference indicates the two signals’ temporal relationship or time delay. The gray area indicates whether the signals are in phase (aligned) at a specific frequency. Positive/negative values indicate the leading signal between dengue and the environmental time series (also represented by arrows in the wavelet coherence diagram).

Figs 6 and 8 display the average cross-wavelet power for cantons belonging to each cluster. These figures highlight the synchronized frequency ranges between the variables, further illustrated by the wavelet coherence, showcasing the time points of synchronization between the signals. The phase difference is also depicted to explain the temporal relationship or time lag between the two signals. The gray area signifies whether the signals are in phase (aligned) at a specific frequency. Positive and negative values indicate the leading signal between dengue and climate/environmental time series, represented by arrows in the wavelet coherence plot.

#### 3.3.1 Clustering analysis of dengue cases and climate variables

Fig 6 depicts the results of the wavelet coherence analysis between dengue cases and the TNA and El Niño 3.4 indices, specifically for the cantons belonging to cluster 4. The results indicate a correlation between dengue and TNA and between dengue and El Niño 3.4 in the 3-yr frequency, except for Puntarenas, where the high-significance area is not clearly identified. Concerning TNA and dengue, the time series demonstrate synchronized movement starting around 2007 and reaching a minimal phase difference between 2010 and 2017. However, the synchronization between El Niño 3.4 and dengue occurs after 2013, with El Niño 3.4 leading by approximately nine months. An annual correlation between dengue and TNA is also observed, whit the leading and lagging time series changing among cantons.

The cantons within this cluster, situated predominantly in the country’s central region near the Pacific Ocean, display coinciding peaks in dengue incidence during 2005, 2010, and 2013. Refer to Fig 6 for a visual representation of this trend. Results for the remaining clusters are in the supplementary material (Figs D-H in S1 Text).

**Fig 7:**
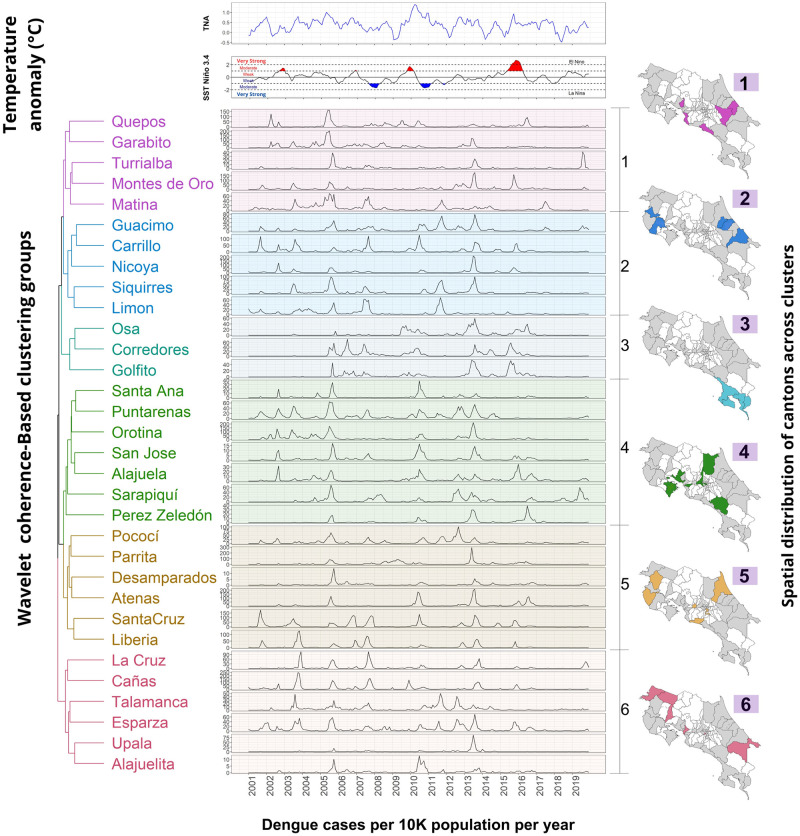
Dengue cases and wavelet coherence-based clustering result for climate indices. This figure summarizes the wavelet coherence-based clustering analysis results. Clusters are represented by a dendrogram, followed by the corresponding dengue time series per 10,000 population for each canton into the cluster and their geographical locations. The top images display the TNA (blue) and El Niño 3.4 (black) time series, with shaded areas indicating values above 1 and below -1. (Shapefiles are publicly available at [60]).

Table 1 summarizes common patterns observed in a specific cluster’s cantons. It is worth noting that while common patterns were easily identifiable in certain groups (such as groups 4, 3, and 5) in all cantons, it must be recognized that not all groups exhibited easily discernible patterns. In Table 1, the term “Time Series” refers to the climate variable that exhibits a correlation with dengue cases. The “Frequency” refers to the dominant frequency of correlation between two time series displayed in the average cross-wavelet power. “Years with significant correlation” refers to an estimated range of years where cantons within the cluster consistently exhibit correlation in the wavelet coherence plots. We use “None” when no correlation is observed in most cantons and “Intermittent” when the correlation is identifiable but does not coincide in the same years. “Leading time series” refer to the time series that predominantly lead in most of the cantons in the phase differences plot. The column labeled “Onset of phase synchronization” indicates the year at which the phase difference value first enters the gray area. Finally, the column labeled “Years with coincident dengue peaks” indicate the years where a peak in cases is observed for all cantons (Fig 7).

**Table 1:** Summary of the common patterns observed between the cantons belonging to each cluster based on the wavelet coherence analysis results between dengue and climate variables.

Cluster	Cantons	Time Series	Frequency	Years with significant correlation*	Leading time series^+^	Lag (Mos.)	Onset of phase sync	Years with coincident dengue peaks
1	Garabito, Matina, Montes de Oro, Quepos, Turrialba	TNA	1-yr	None	Dengue	0–3	2005	2005
		El Niño 3.4	3-yr	None	El Niño 3.4	9	2011**	
2	Carrillo, Guacimo, Limón Nicoya, Siquirres	TNA	1-yr	2010–2016	Dengue	0- 3	2001	2005
		El Niño 3.4	1.5, 2 yr	Intermittent	Dengue	-		
3	Osa, Corredores, Golfito	TNA	1 yr	2012–2017	Dengue	0–3	2004	2005, 2010, 2013
			3 yr	2008–2015	Dengue	0–3	2007	
		El Niño 3.4	3 yr	2008–2017	El Niño 3.4	0–9	2007	
4	Alajuela, Orotina, Pérez Zeledón, Puntarenas San José, Santa Ana, Sarapiquí	TNA	1 yr	2012–2017	Dengue	0–3	2001	2005, 2010, 2013
			3 yr	2008–2013	Both	0	2007	
		El Niño 3.4	3 yr	2007–2017	El Niño 3.4	9	2013	
5	Atenas, Desamparados, Liberia, Parrita, Pococí, Santa Cruz	TNA	1 yr	2012–2017	TNA	0–3	2001	2005, 2013
			3 yr	None	TNA	0–9	2008	
		El Niño 3.4	3 yr	Itermittent	El Niño 3.4	0–9	2013	
6	Alajuelita, Cañas, Esparza, La Cruz, Talamanca, Upala	TNA	1 yr	2012–2017	TNA	0–3	2004	2005, 2013
			3 yr	None	TNA	0–9	2008	
		El Niño 3.4	3 yr	None	El Niño 3.4	0–9	2013	

*Report estimated range of years during which all cantons within the cluster consistently exhibit a notable area of high significance in the wavelet coherence plots.

^+^ Refer to the time series that predominantly lead in most cantons in the phase differences plot.

**Except Matina and Turrialba.

#### 3.3.2 Clustering analysis of dengue cases and local environmental variables

**Fig 8:**
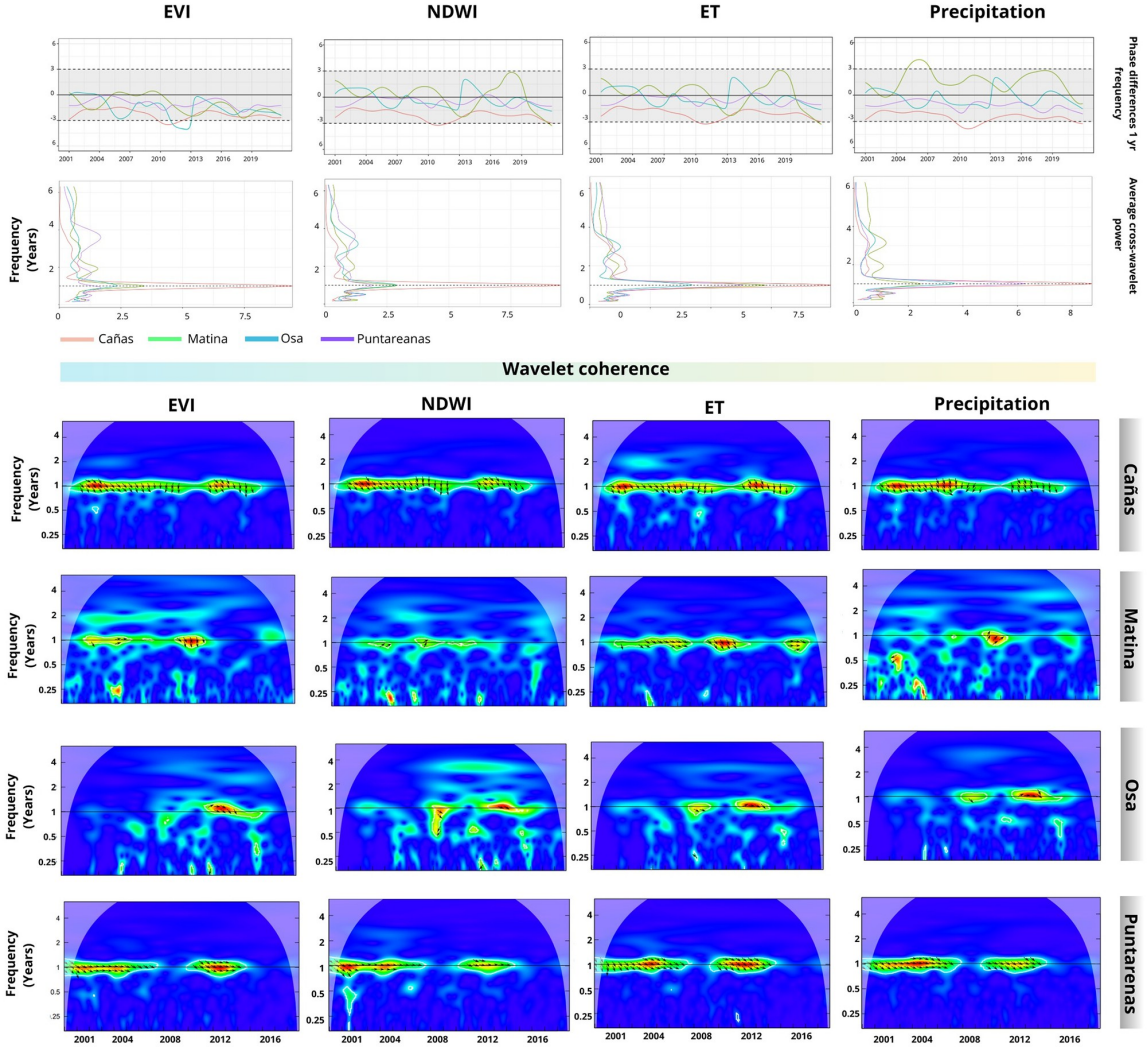
Wavelet coherence results cluster 5. The average cross-wavelet power (top-left) highlights the synchronized frequency in years, further illustrated by the coherence of the wavelets that also display the synchronization time points between the signals. The phase difference indicates the two signals’ temporal relationship or time delay. The gray area indicates whether the signals are in phase (aligned) at a specific frequency. Positive/negative values indicate the leading signal between dengue and the environmental time series (also represented by arrows in the wavelet coherence diagram).

**Fig 9:**
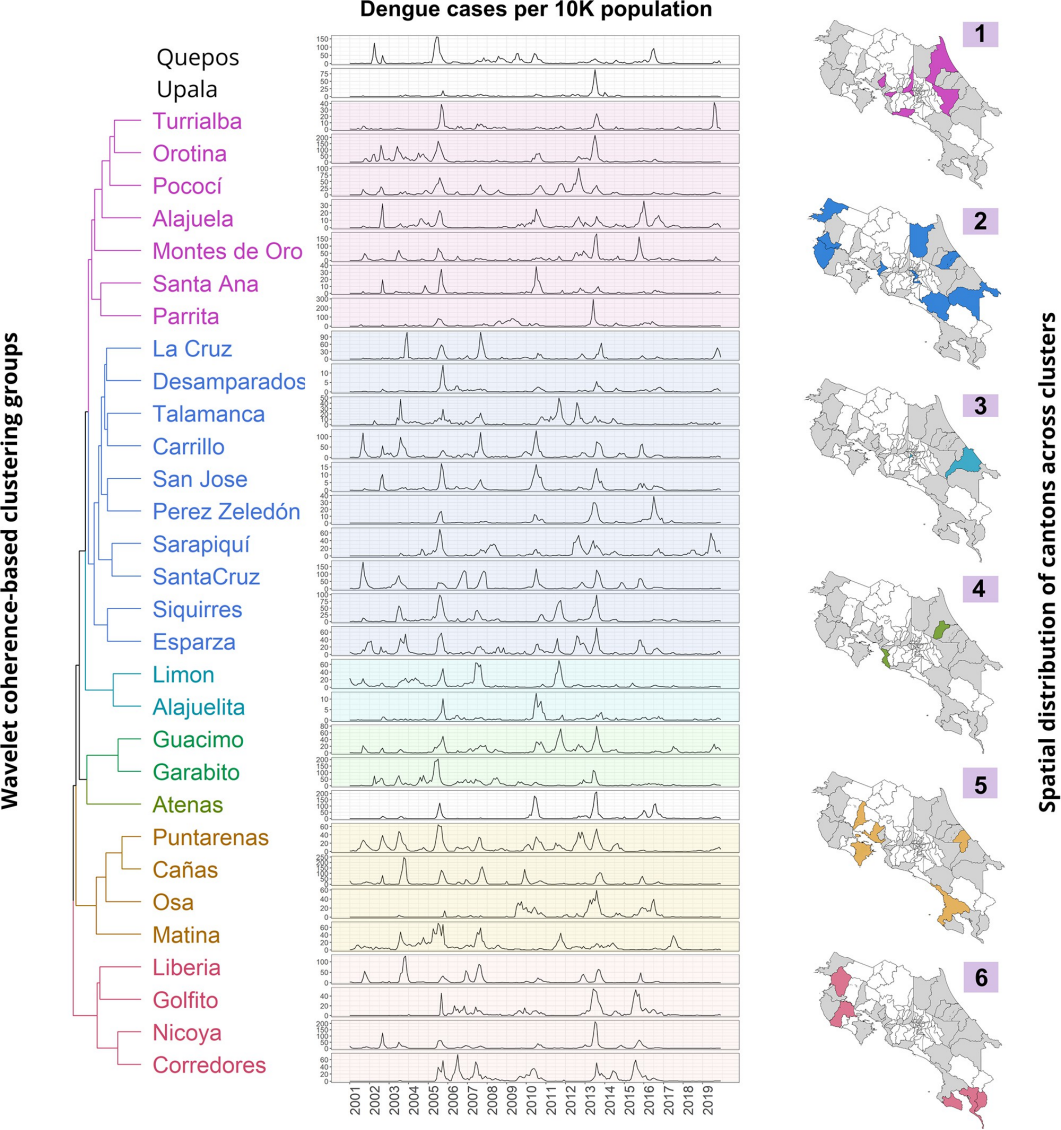
Dengue cases and wavelet coherence-based clustering result for environmental indices. The dendrogram illustrates the clusters that have been identified. Adjacent to the dendrogram, a graph presents the dengue time series per 10,000 inhabitants for each cluster over time. This is followed by the time series for each of the environmental variables. On the right side, maps indicate the geographical locations of the identified clusters. (Shapefiles are publicly available at [60]).

Fig 8 presents the correlation between dengue cases and four local environmental indices (EVI, NDWI, ET, and Precipitation) in the cantons belonging to cluster 5. The correlation is observed in the 1-yr frequency across all cantons. The phase difference values indicate that the analyzed time series are in phase, with EVI consistently leading in all cantons. However, the remaining variables’ leading and lagging time series vary. An annual correlation is observed over time in Cañas and Puntarenas. However, although the correlation is observed in Matina and Osa, it is not as persistent and straightforward as in Cañas and Puntarenas. On the other hand, the dengue time series peaked around 2005 and 2013 in all cantons (Fig 9). The results for the remaining cantons can be found in the supplementary material, specifically in Figs I-M in S1 Text.

The wavelet coherence cluster analysis successfully identified distinct groups of cantons. However, uncovering common patterns among cantons within the same cluster proved challenging. Nevertheless, it is important to highlight that in Cluster 1, ET and precipitation lead by less than three months for all cantons except Parrita. In Cluster 2, all environmental variables lead by less than three months, except in Sarapiquí, Talamanca, and Pérez Zeledón. In Cluster 3, all environmental variables lead after 2011 in Alajuela. In Cluster 4, EVI and ET lead consistently over time in Guacimo. Lastly, in Cluster 6, all variables lead by less than three months in Liberia and Nicoya.

During the clustering process, Atenas, Upala, and Quepos remained isolated as unique cantons within their respective groups. Quepos, a tourist destination situated in the central Pacific, experiences abundant rainfall. The wavelet coherence analysis revealed a brief period of significant correlation solely between dengue cases and ET and precipitation, with the dengue time series leading the relationship (Fig N in S1 Text). Upala, located in the northern region with a climate classified as rainy with monsoon influence, exhibited a substantial region of high joint significance from 2012 to 2015 (coinciding with the worst dengue outbreak in Upala in 2013) with all variables except EVI. The dengue time series acted as the leading time series, except in the coherence with ET, where the time series demonstrated a vanishing phase difference (Fig N in S1 Text). Atenas, situated in the Central Valley, displayed two frequencies of joint significance within one year, with a disruption observed between 2007 and 2009. The time series showed vegetation variables leading, except from 2009 to 2013 when the dengue time series became the leading time series. The lag shifted between 0–3 months (Fig N in S1 Text).

In contrast to other areas, it is worth mentioning that Talamanca only correlates with ET in a short period. Talamanca is renowned for its rich cultural heritage and significant indigenous population, which has diligently preserved its traditional way of life, customs, and languages. This indigenous population brings a unique perspective and knowledge to the region, including their understanding of natural remedies and treatments for various diseases, which may affect official dengue reports in the canton.

## 4 Discussion

In this study, we employed a wavelet approach to investigate the correlation patterns between dengue cases and various climate and local environmental variables in 32 cantons of interest for the Minister of Health in Costa Rica. The analysis encompassed nearly two decades of data, providing insights into the dynamics between these factors.

The intricate relationships between ocean/climate indices and local climate/environmental variables hold significant potential to influence dengue transmission dynamics. While ocean indices such as TNA and ENSO may not directly impact dengue incidence, their effects can be mediated through complex pathways involving intermediate factors. Changes in ocean temperatures can trigger shifts in atmospheric circulation patterns, subsequently impacting regional climate conditions. For instance, ENSO can induce alterations in precipitation patterns, leading to variations in water availability, humidity, and breeding habitats for dengue vectors. Additionally, modifications in sea surface temperatures can influence temperature gradients, affecting air temperature and subsequently impacting vector survival rates and dengue virus replication within vectors. Higher temperatures can accelerate mosquito development, enhance survival rates, and shorten the incubation period of the dengue virus inside the mosquito, thereby increasing the potential for transmission. Similarly, the distinct wet and dry seasons play a crucial role. Rainfall creates suitable breeding sites for mosquitoes, thus intensifying vector densities and transmission risks during the wet season. However, the dry season can also contribute to dengue transmission, as people often store water in containers that can serve as breeding grounds for mosquitoes. Furthermore, variations in ocean indices may trigger changes in vegetation dynamics and land surface properties, influencing environmental indicators like NDVI and NDWI, which can indirectly impact vector habitats. Therefore, understanding the interplay between ocean/climate indices and local climate/environmental variables is crucial for unraveling the intricate mechanisms that govern dengue transmission dynamics and informing effective public health interventions.

### 4.1 Climate variables

The analysis of wavelet coherence reveals that multiannual dengue frequency (3-yr) is correlated with climate variables (TNA and El Niño) in cantons mainly located in the North and South Pacific coast and cantons in the center of Costa Rica. These regions share certain common characteristics. The North Pacific region falls within the Pacific precipitation regime, known for its distinct dry and rainy periods [48]. Similarly, the geographical features in the country’s center and the South Pacific region create a climate with a short but favorable dry period, followed by intense rainfall [48]. Severe drought years in these regions, except for 2001, have coincided with El Niño events [49]. A study conducted by Cuong et al. [19] found that dengue incidence during the dry season contributed to approximately 63% of the variability in the magnitude of dengue epidemics, suggesting that the dry season plays a significant role in shaping the magnitude of dengue epidemics.

The findings unveiled that within cantons demonstrating a correlation between dengue cases and climate variables in the 3-yr period, the TNA and El Niño time series exhibited a leading role over dengue cases, with a lag of approximately nine months. This result implies that alterations in climate conditions could potentially act as a precursor to dengue cases in those specific cantons. The correlation observed over the defined period aligns with the periodicity of El Niño, which typically occurs every 3–7 years and lasts for an average duration of 9–12 months [27]. Previous studies [11, 13] have also reported a delayed effect of climatic variables on dengue incidence, highlighting similar patterns and temporal associations between dengue cases and El Niño events.

The correlation between TNA and dengue cases demonstrates regional variability. Specifically, the Pacific and central regions of the country exhibit a significant correlation at the 3-yr frequency, while no notable correlation is found in the Caribbean region. This distinction in correlation can be related to the interaction between El Niño and TNA and its impact on the distinct climatic conditions prevailing in each region. Although there is no established statistical association between sea surface temperature anomalies in the two oceans, the concurrent interaction of both anomalies has been observed to influence seasonal precipitation. The dry conditions typically observed on the Pacific slope during warm-phase ENSO years are less severe when the Caribbean region experiences warmer temperatures [50]. Conversely, the Caribbean region encounters intense rainy scenarios during El Niño events [49]. Consequently, the correlation between TNA and dengue cases may be less apparent in the Caribbean due to the abundant rainfall, which can suppress mosquito populations and reduce dengue transmission.

The analysis showed patterns underscore that weather is not the only driver of dengue cases. Some findings include correlations between dengue cases and climate variables at 1, 1.5, and 2 years. In most cases, the dengue time series consistently preceded fluctuations in climate variables. However, dengue cases influencing climate variables are a biologically unlikely relationship. It is more plausible that changes in El Niño 3.4 and TNA increase subsequent dengue transmission [51], given that the anomalies in the ocean temperatures affect local temperature and precipitation, which are directly associated with the virus ecology and transmission. On the other hand, dengue data shows that 2005 and 2013 stood out with notably elevated dengue case counts compared to historical data. The wavelet analysis results showed a correlation between dengue cases and climatic variables around the year 2013 within specific cantons. However, this correlation pattern is not observed around the year 2005. These finding underscores the significance of multifaceted drivers behind dengue dynamics beyond climate variables. Factors like population mobility, population immunity to circulating serotypes, healthcare infrastructure, and interventions play pivotal roles in shaping the temporal dynamics of dengue outbreaks [52, 53].

The clustering analysis reveals distinct clusters of cantons that exhibit similar patterns regarding the impact of climatic variables and the initiation of time series synchronization. This information holds significant value for public health authorities as it allows them to identify regions with comparable vulnerability to dengue transmission. Consequently, it enables the implementation of targeted interventions and proactive measures within the next nine months following an El Niño phenomenon in those areas. By leveraging this knowledge, public health agencies can allocate resources and focus efforts to mitigate the potential risks associated with dengue outbreaks in the identified regions.

### 4.2 Environmental variables

The wavelet coherence analysis conducted in this study revealed a statistically significant correlation between the annual dengue frequency with local environmental variables. The analysis identified persistent correlation over time in cantons, primarily in the North Pacific and the center of the country’s Northwest region. These cantons fall within the tropical and subtropical climate zones of Costa Rica. As a result, they experience relatively high temperatures throughout the year with distinct wet and dry seasons that provide favorable conditions for mosquito vector proliferation. Furthermore, the cantons in the metropolitan area of Costa Rica, which is situated in the country’s central region, have relatively high population densities. This higher concentration of people increases the likelihood of human-mosquito interactions, providing more opportunities for transmitting the dengue virus.

Although the wavelet coherence cluster analysis successfully identified distinct clusters of cantons by incorporating local environmental variables, extracting shared patterns among cantons within each cluster proved challenging. This suggests that the clustering approach may have limitations in accurately capturing the similarities and associations of environmental variables. To gain a more comprehensive understanding of these relationships, future research should explore alternative methods and approaches that can better uncover and analyze the shared patterns and associations among cantons within each cluster. On the other hand, the inability to identify consistent patterns between cantons may also be attributed to the country’s vegetation characteristics. Costa Rica boasts bountiful and varied vegetation thanks to its tropical climate and diverse topography. The nation has a vast rainforest, cloud forests, and numerous other ecosystems providing a nurturing environment for thriving plant life. This lush vegetation provides ideal conditions for the proliferation of the *Aedes aegypti* mosquito.

Climate and environmental variables exhibited significant coherence with dengue cases in various geographically diverse regions of Costa Rica. However, it is important to note that spatial heterogeneity in the effects of these variables exists, as has also been observed in other regions of the world [12, 52]. It is important to consider that changes in the periodicity over time may also be to other external factors or inherent characteristics of the disease. Alternative explanations for the multi-annual cycles of dengue epidemics have been proposed, such as partial cross-immunity among the four serotypes of the dengue virus [54]. Additionally, sociological factors such as population structure, unplanned urbanization, and international transportation of infected people and mosquitoes may also affect dengue transmission [55].

According to the results, climate variables may be significantly more influential in designing accurate mathematical models for predicting dengue outbreaks and developing effective public health interventions in Costa Rica. Nevertheless, the intricate interplay among biological, socioeconomic, environmental, and climatic factors introduces significant spatiotemporal heterogeneity in the intensity of dengue outbreaks. Recognizing and comprehending this heterogeneity in dengue transmission holds the potential to enhance epidemic prediction and bolster disease control efforts. By delving into the complexities of these interactions, researchers and public health authorities can gain a deeper understanding of dengue transmission dynamics, ultimately leading to more effective strategies for epidemic prediction and control.

Previous studies have proposed predictive models for assessing the impact of climatic and vegetation variables on a country’s dengue fever incidence. Fuller et al. [56] introduced a straightforward structural model integrating lagged sea surface temperature (SST) and MODIS vegetation indices. Their model accounted for 83% of the variability in weekly cases of dengue fever and dengue hemorrhagic fever (DF/FHD) in Costa Rica. However, an analysis conducted at the cantonal level revealed spatial variations in the influence of climate and environmental factors on dengue case incidence. This finding suggests that the local-scale impact of these variables on dengue transmission may deviate from global expectations. Consequently, localized predictions are valuable for guiding intervention strategies and resource allocation.

Additional research demonstrates that incorporating the same climatic variables analyzed in our study to predict relative risk areas for dengue outbreaks in all cantons may result in model projections failing in certain locations. This observation, as demonstrated by [57–59], emphasizes the significance of comprehending the local correlations between dengue cases and external factors such as climate and socioeconomic drivers. Such understanding can lead to improved predictions of dengue locally, enabling better-informed decision-making regarding interventions and resource allocation.

Many studies have been conducted in tropical and subtropical regions to elucidate the complex interactions between climate variables and dengue transmission [11–13]. However, the localized analysis makes this study different, considering other variables such as TNA, EVI, NDWI, evapotranspiration, and precipitation.

Wavelet analysis allows a retrospective study to characterize outbreaks over time, which provides important guidelines for future modeling approaches in which explicit mechanisms can be incorporated. However, as climate factors are not the only predictors influencing the rise in dengue infection, future studies must include other factors unique to this area, such as the predominant circulating dengue viruses, anthropogenic factors, and herd immunity.

## Supporting information

S1 Text (PDF)
