# Supplementary material for: Common patterns between dengue cases, climate, and local environmental variables in Costa Rica: A wavelet approach
Source: PLOS Glob Public Health. 2023 Oct 19;3(10):e0002417. doi: 10.1371/journal.pgph.0002417 (PMC10586647; doi:10.1371/journal.pgph.0002417)
Supplement: S1 Text — (PDF) [file pgph.0002417.s001.pdf]

Supplement: Common patterns between dengue cases, climate, and local environmental variables in Costa Rica: a wavelet approach

Yury E. García, Shu Wei Chou-Chen, Luis A. Barboza, Maria L. Daza–Torres, J. Cricel

1 Dengue cases behaviour in the 32 cantons of interest in Costa Rica

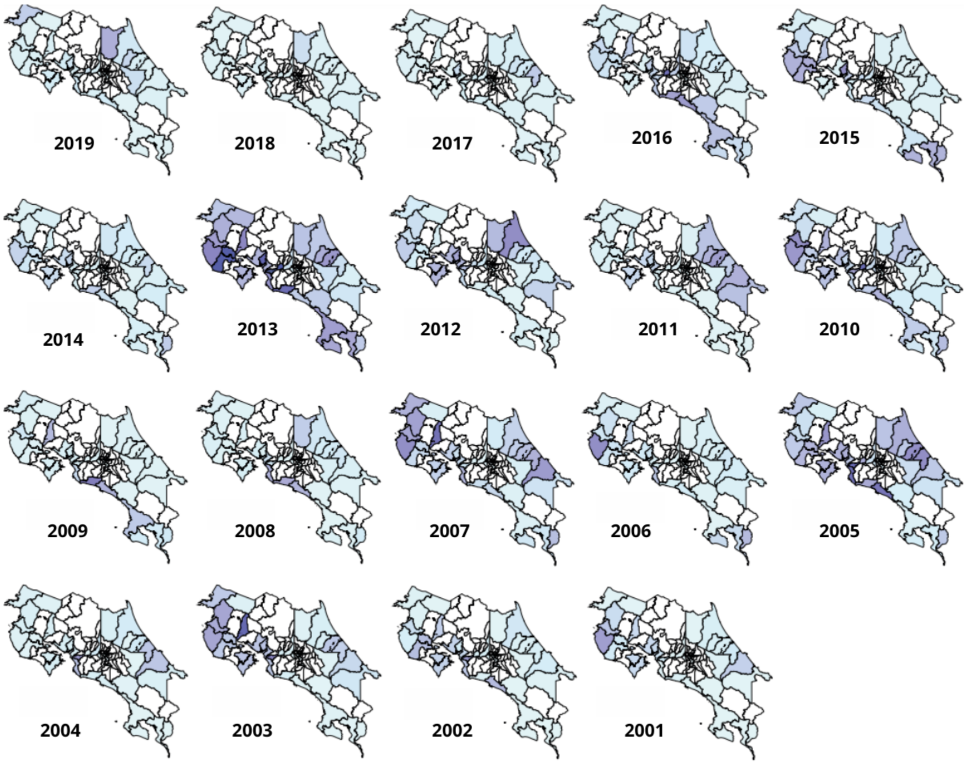

Figure A. Temporal distribution of dengue cases across geographical regions. The figure illustrates the number of dengue cases per 10,000 population over time in the 32 selected cantons of Costa Rica analyzed in this study. The white-colored cantons were excluded from the study.(Shapefiles are publicly available at (1))

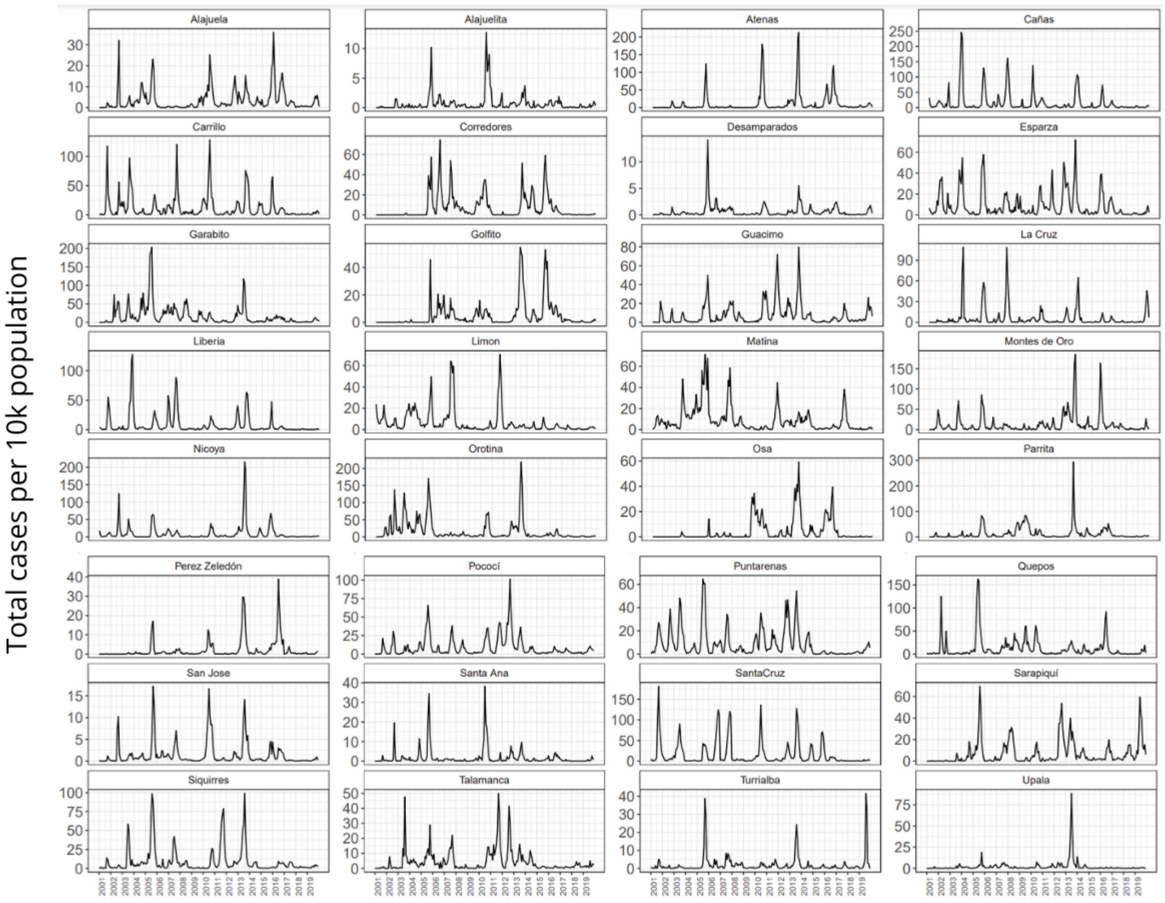

Figure B. Dengue cases over time per canton. Dengue cases per 10,000 population over time in the 32 cantons of interest for this analysis in Costa Rica.

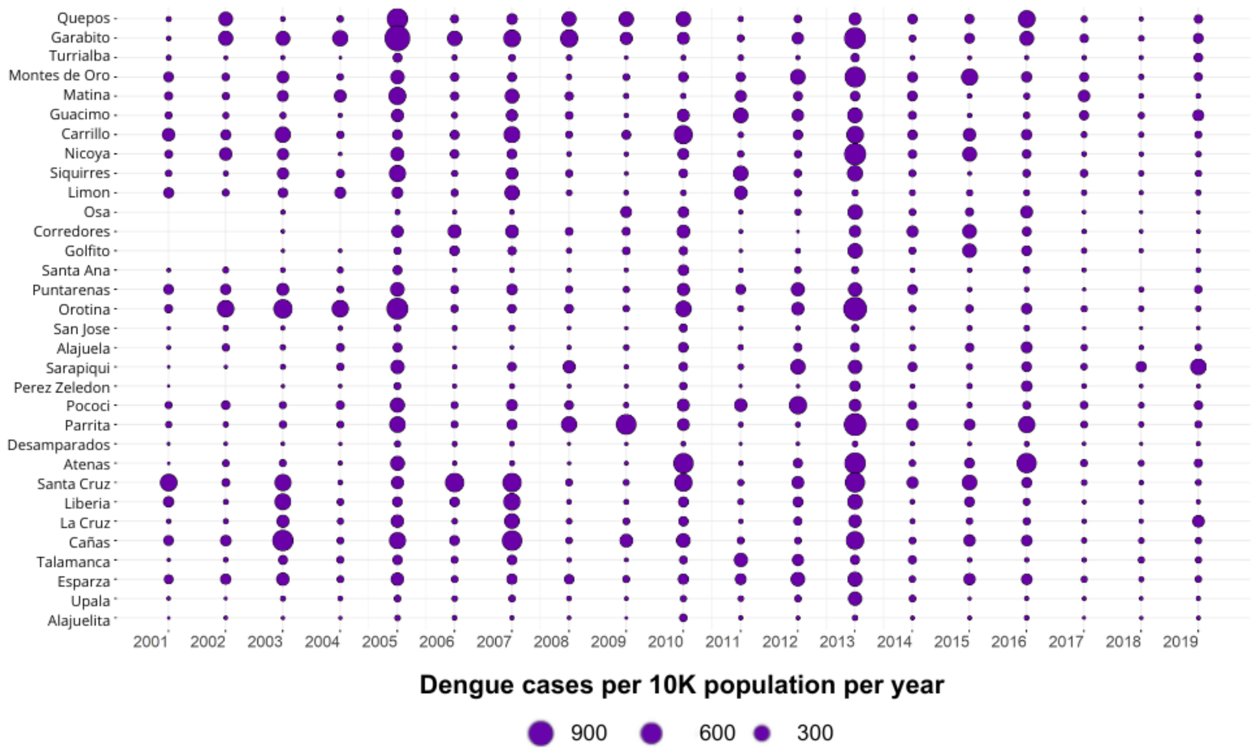

Figure C. Dengue cases per 10,000 population over time per canton. The bubbles on the graph represent the total number of cases per 10,000 population per year in the 32 selected cantons of interest for this analysis in Costa Rica. Each bubble size corresponds to the relative magnitude of dengue cases in each canton, providing an overview of the disease burden in the study area.

2 Considerations for interpreting the results

We employed the R-package "WaveletComp version 1.1" (3) to conduct our wavelet time-series analyses. This package offers a range of algorithms and tools for visualizing and interpreting correlations in time series data. To assess the significance of a wavelet power spectrum, it is compared to simulated or theoretical spectra representing a null hypothesis. In our analysis, the null hypothesis assumed "no periodicity". To test this hypothesis, we employed 1000 bootstrap series using surrogate time series with a similar spectrum, specifically autoregressive (AR) time series (3, 4). This approach allowed us to determine the significance levels robustly and derive meaningful interpretations from the wavelet analyses.

To visualize wavelet coherence, an image plot is commonly employed, where the color bar represents the magnitude of the power gradient. This technique effectively identifies areas of statistical significance, indicating significant shared periods between the analyzed time series. In our analysis, we utilized quintiles to split the power levels, ensuring a more balanced distribution of colors in the images. However, it's important to note that this approach may amplify artifacts (3).

In the wavelet plots, statistically significant regions are highlighted by a solid black line, indicating rejection of the null hypothesis at the default significance level of 10%

(p-values below 0.1). These regions represent areas where the coherence between the time series is deemed significant. Additionally, the cones of influence, which represent the regions where edge effects increase the uncertainty of the analysis, are depicted as lighter-shaded regions. These cones of influence help account for the potential impact of boundary effects on the wavelet analysis results (3).

The phase difference and time lag between two-time series, denoted as $x(t)$ and $y(t)$, provide insights into their synchronization and temporal relationship. These measures can be obtained using wavelet analysis and are useful for understanding the phase dynamics between the series (2). To visualize the phase differences, they can be displayed as arrows within the white contour lines to indicate their significance and prevent artifacts.

Horizontal arrows pointing to the right indicate that the two series, $x(t)$ and $y(t)$, are in phase at the respective period, with a vanishing phase difference. Conversely, horizontal arrows pointing to the left indicate that the two series are out of phase. The magnitude of the phase difference expressed as an absolute value, determines whether the two series are moving in or out of phase. A value smaller (larger) than $\pi/2$ indicates that the two series are moving in phase (out of phase), respectively, at the specific frequency or period being considered. The sign of the phase difference indicates which series is leading in this relationship (3).

In our analysis, angles between $0°$ and $90°$ for the arrows signify that the leading time series is dengue. In contrast, angles between $270°$ and $360°$ indicate that the climatic or environmental variables take the lead. This information helps in understanding the directional relationship between the variables. Furthermore, the average power provides insights into significant periods across time, highlighting frequency ranges that exhibit significant correlations (3).

3 Clustering analysis

For each cluster we present:

- **Average cross-wavelet power**: To identify the significant periods that are also displayed in the wavelet coherence.
- **Phase difference:** provides valuable insights into the synchronization and lag between time series. Represented by the arrows in the wavelet plots.
- **Wavelet coherence**: The wavelet coherence representation employs colors to depict increasing power intensity, ranging from blue to red. The 95% confidence levels are indicated by white lines, and shaded areas indicate the presence of a significant edge effect. The y-axis represents periodicity, while the x-axis represents time.

3.1 Clustering analysis of dengue cases and climate variables

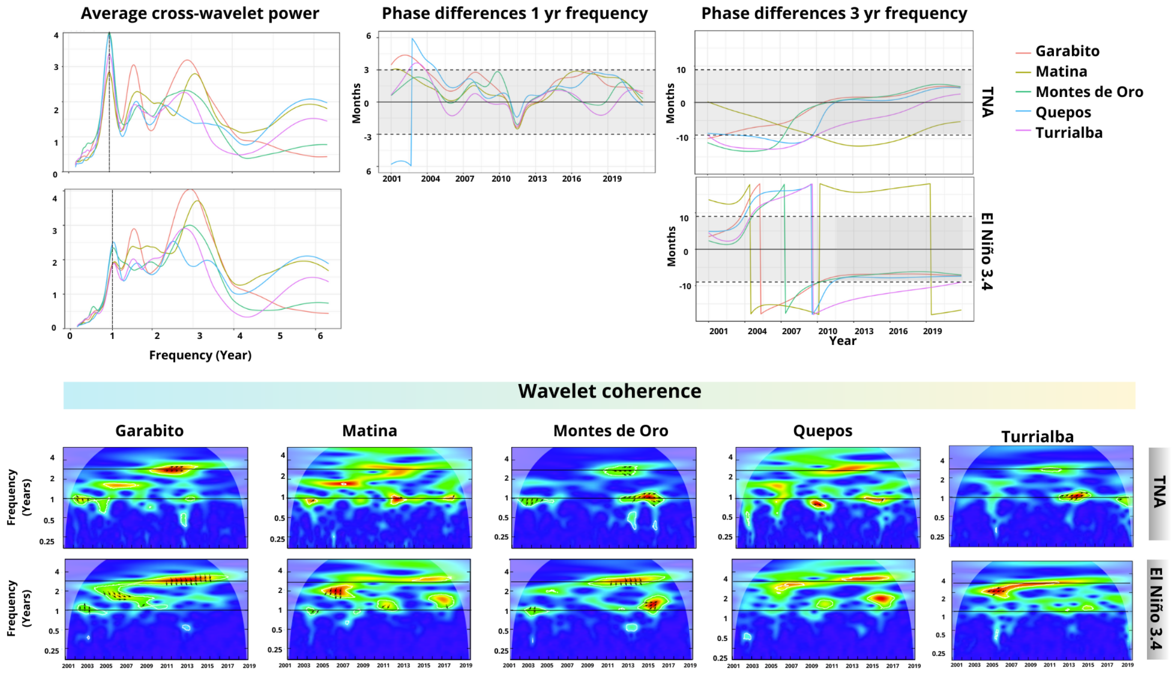

Figure D. Cluster 1. The dominant period of synchronization is annual in both cases, between dengue and TNA, and between dengue and El Niño 3.4. Additionally, synchronization is observed at approximately 1.5 years and around 3 years, although this pattern is not consistently observed across all cantons. Specifically, the synchronization between TNA and dengue cases within the 3-year period begins after 2010, except for the time series in Matina. They exhibit complete synchronization for a brief period before dengue cases take the lead. In the case of dengue cases and El Niño 3.4, they started moving in phase after 2011 in all cantons except Matina and Turrialba, with El Niõ 3.4 ahead by roughly 9 months. Areas of significance in the 3-year period for both climate variables are identified solely in Garabito and Montes de Oro.

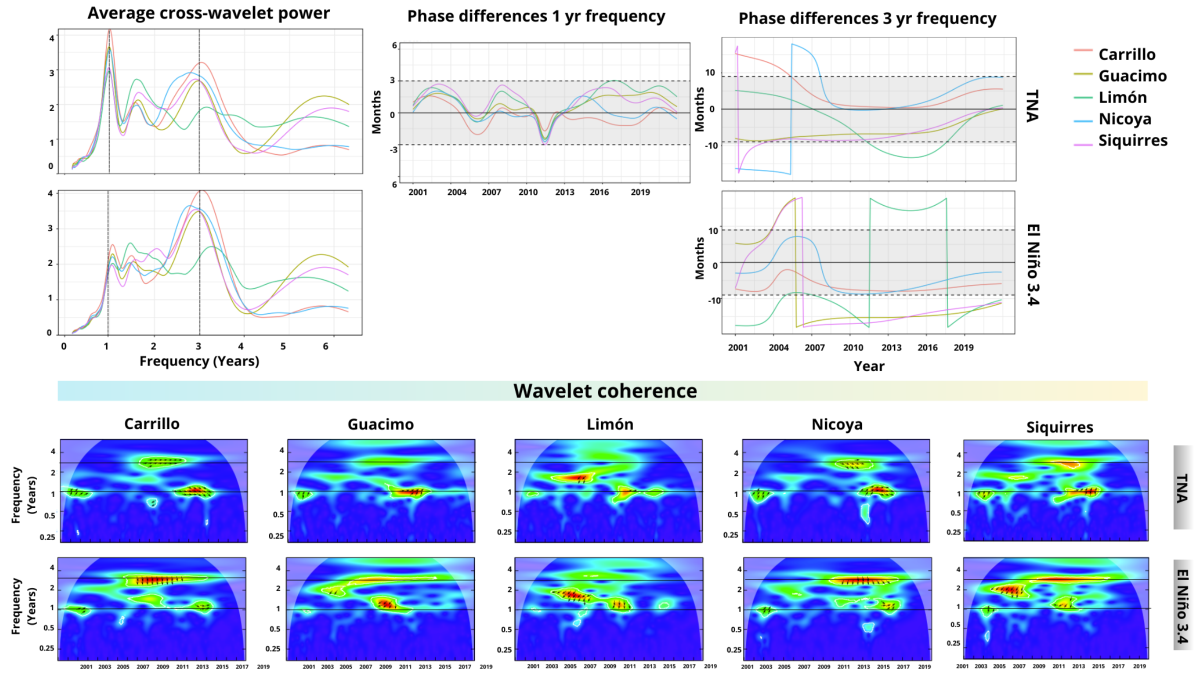

Figure E. Cluster 2. The dominant synchronization periods between dengue and TNA occur annually and within a 3-year period (excluding Limón). Within a year, the time series move in phase, with dengue leading most of the time. Starting from 2007, the time series began moving in phase within the 3-year period, although the dominant time series varied among cantons. Similarly, for dengue cases and El Niño 3.4, the dominant synchronization periods are observed around 1, 2, and 3 yr (excluding Limón). Within the 3-yr period, some cantons exhibit out-of-phase movement, while others show in-phase synchronization. Notably, areas of significance between dengue and TNA, as well as between dengue and El Niño 3.4, are identified in the 3-yr period specifically in Carrillo and Nicoya.

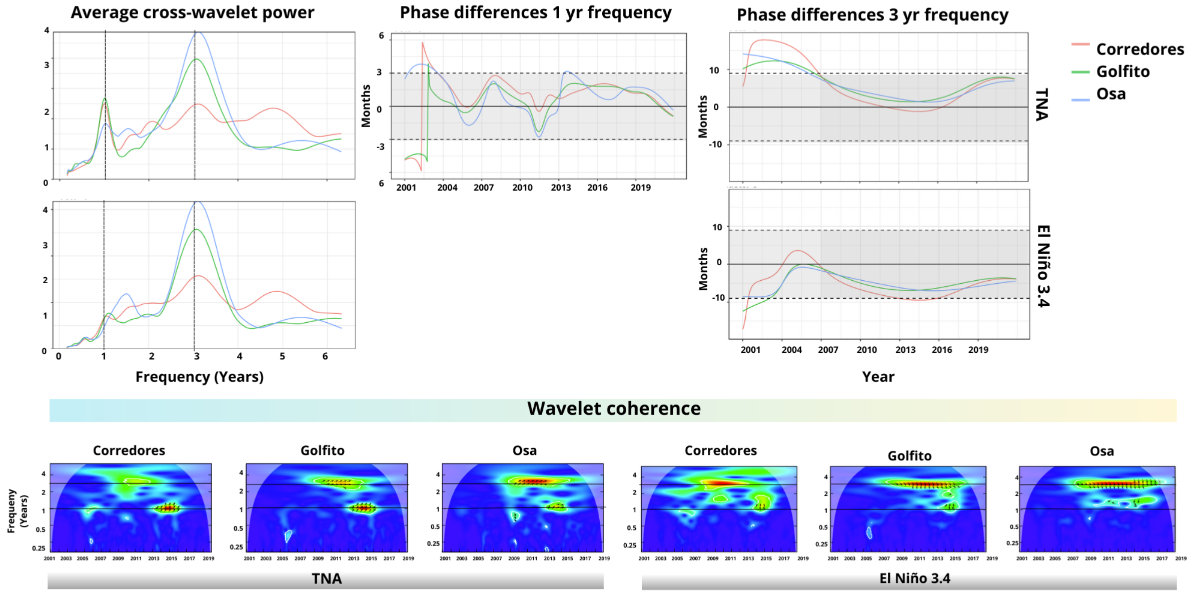

Figure F. Cluster 3. The dominant periods of synchronization between dengue and TNA occur annually and within the 3-yr period.

In the 1 yr period, the time series generally move in phase, with dengue leading most of the time and an area of high significance in all cantons around 2012-2017. In the 3-yr period, starting from 2007, the time series began moving in phase, with dengue leading most of the time too. An area of high significance is identified in the 3-yr period around 2008-2015, in all cantons. In the case of dengue cases and El Niño 3.4, the dominant synchronization period is also observed at 3 yr period. After 2007, the time series move in phase, with El Niño 3.4 leading by 0 to nine months. An area of high significance is observed in all cantons during a period estimated to be around 2008 to 2017.

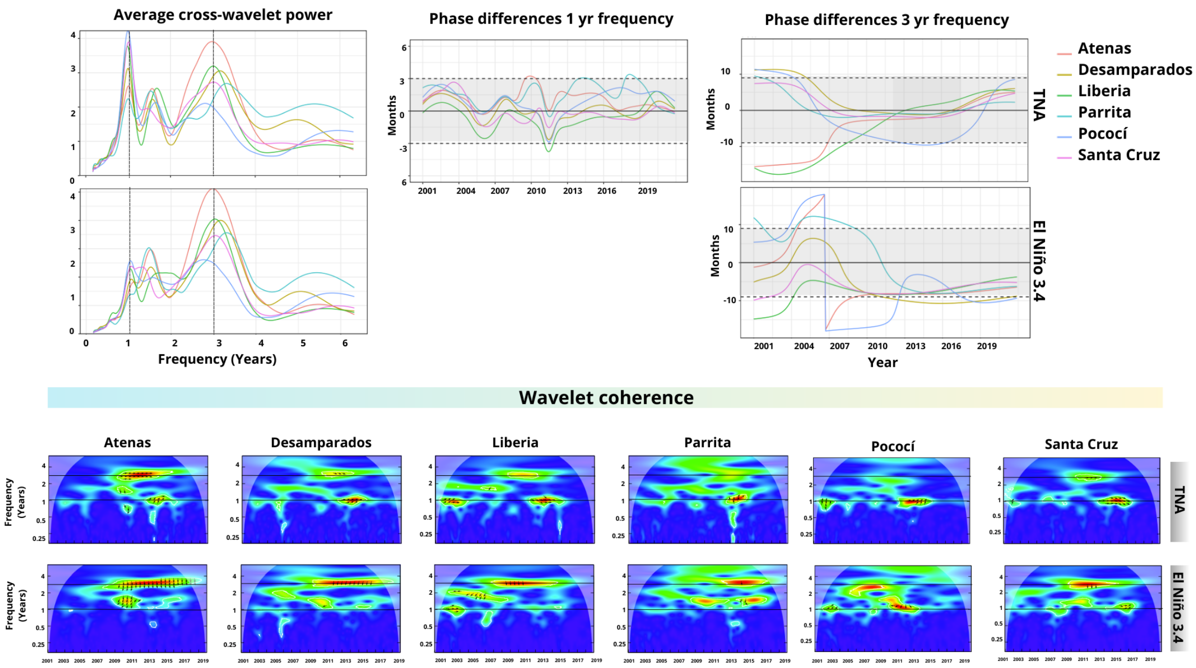

Figure G. Cluster 5. The dominant synchronization periods between dengue and TNA primarily occur in the 1-yr period where the time series move in phase, with dengue leading in most cantons consistently over time. Notably, an area of high significance is identified in all cantons between 2012 and 2017. TNA and dengue also are synchronized in the 3-yr period in most of the cantons. Time series synchronization began around 2008, with the time series moving in phase and TNA leading by less than 3 months before 2017. However, no significant correlation area is observed for all cantons in the wavelet coherence plot. It is identified only for data in Atenas.

When examining dengue cases and El Niño 3.4, the dominant synchronization period is observed in the 3-yr period for most cantons, with the time series moving in phase after 2013. However, it should be noted that the time series in Desamparados does not exhibit this synchronization pattern. The areas of high significance are particularly pronounced in Atenas, Desamparados, and Liberia.

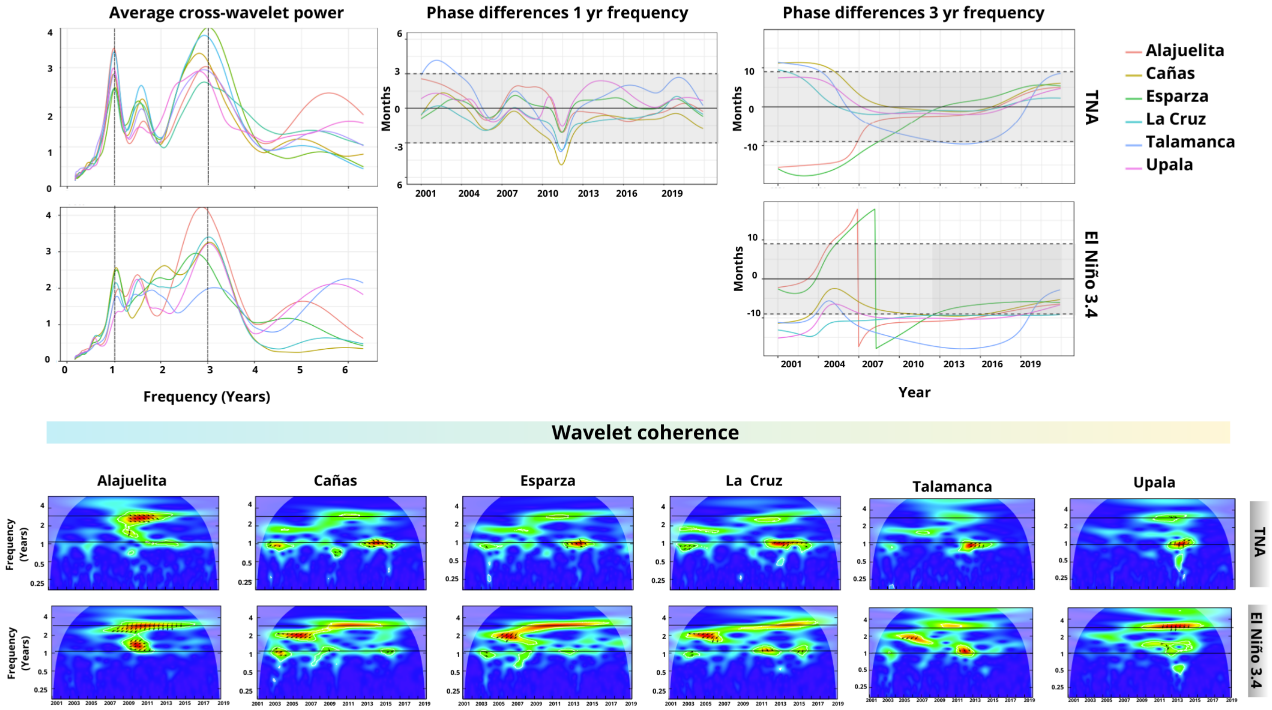

Figure H. Cluster 6. The dominant synchronization periods between dengue and TNA are observed in the 1, 1.5, and 3 yr period. However, areas of high significance are in the wavelet plot are only identified in the 1-yr period for all the cantons, except for Alajuelita, where an area of high significance is explicitly observed in the 3-yr period. In the 1-year period, the time series are in phase, with dengue leading as the dominant time series. However, in the 3-year period, synchronization begins after 2008, with TNA leading most of the time.

The synchronization periods for dengue cases and El Niño vary across all cantons, primarily around 1, 1.5, and 3 years. The phase difference indicates that the time series are in phase after 2013, where El Niño leads by approximately 9 months, except for Talamanca. However, the areas of high significance in the wavelet coherence analysis vary among cantons and change over time.

3.2 Clustering analysis of dengue cases and local environmental variables

The following plots present clusters based on dengue cases and local environmental variables. Identifying common patterns between cantons belonging to the clusters has proven to be challenging. However, all-time series show correlation primarily within the 1-year period, with the time series moving in phase.

In Cluster 1, it is worth noting that ET and precipitation lead by less than 3 months for all cantons except Parrita. In Cluster 2, all environmental variables lead by less than three months, except in Sarapiquí, Talamanca, and Pérez Zeledón. Moving on to Cluster 3, in Alajuela, all environmental variables lead after 2011. In Cluster 4, Guácimo shows that EVI and ET lead consistently over time. Lastly, in Cluster 6, all variables lead by

less than three months in Liberia and Nicoya.

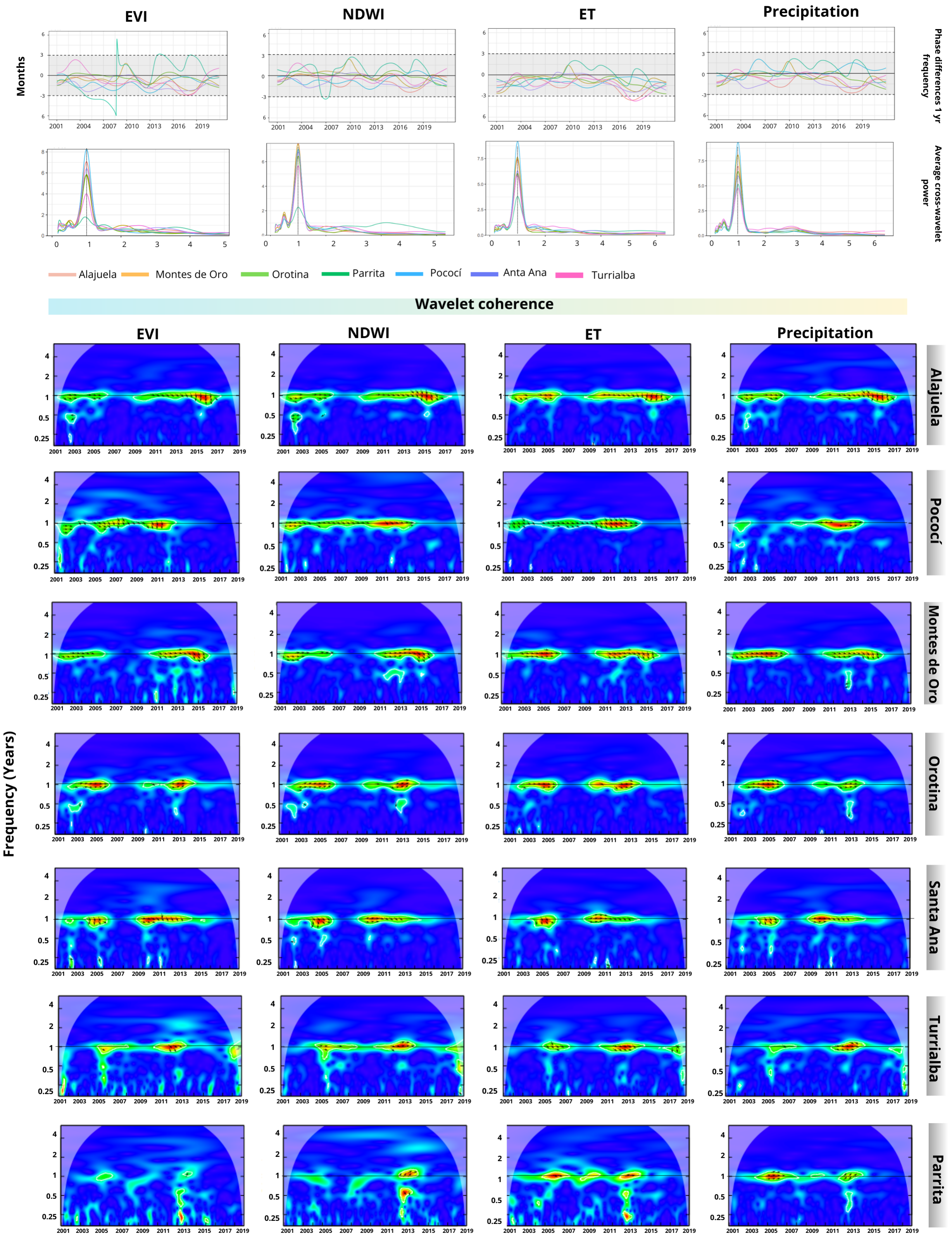

Figure I. Cluster 1.

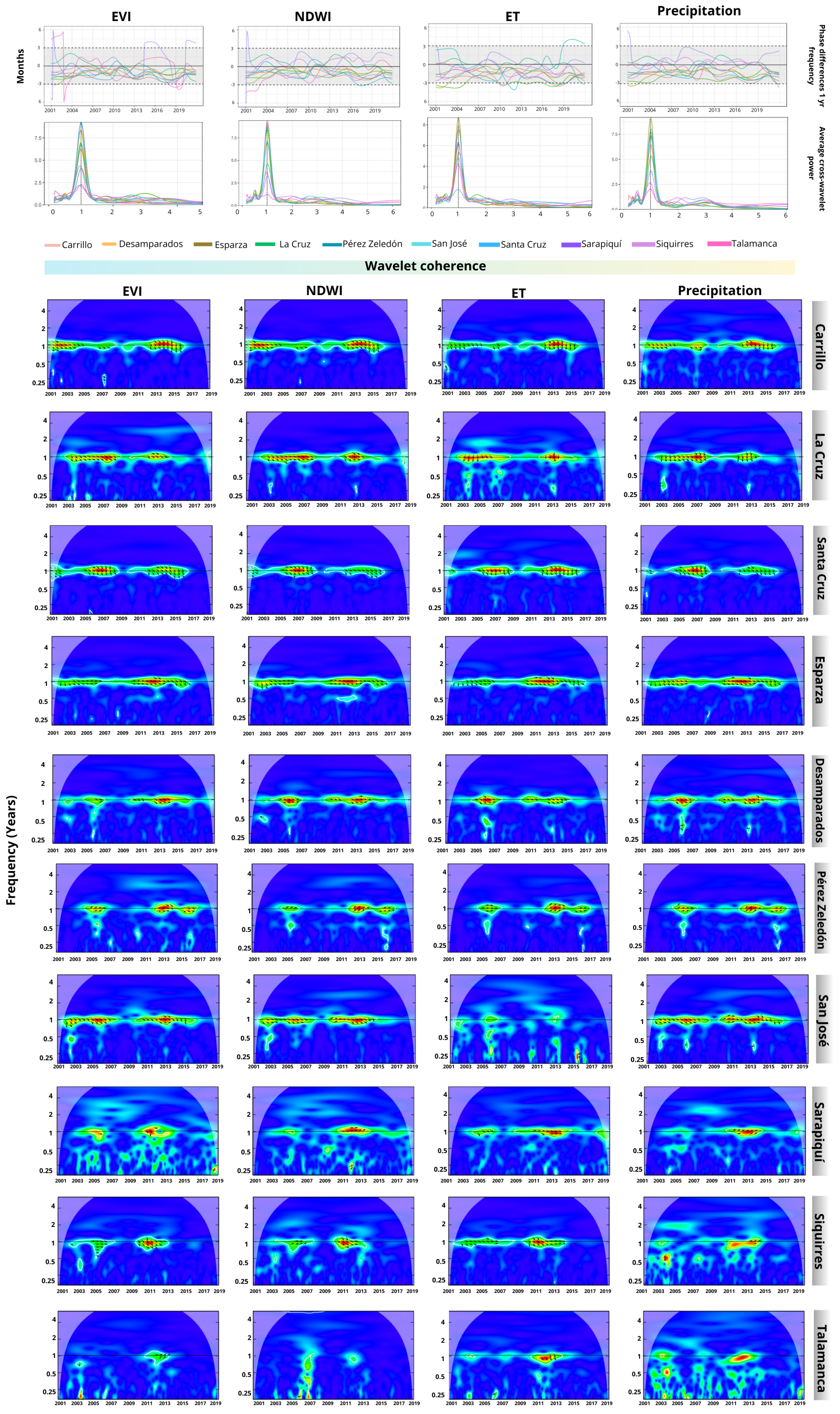

Figure J. Cluster 2.

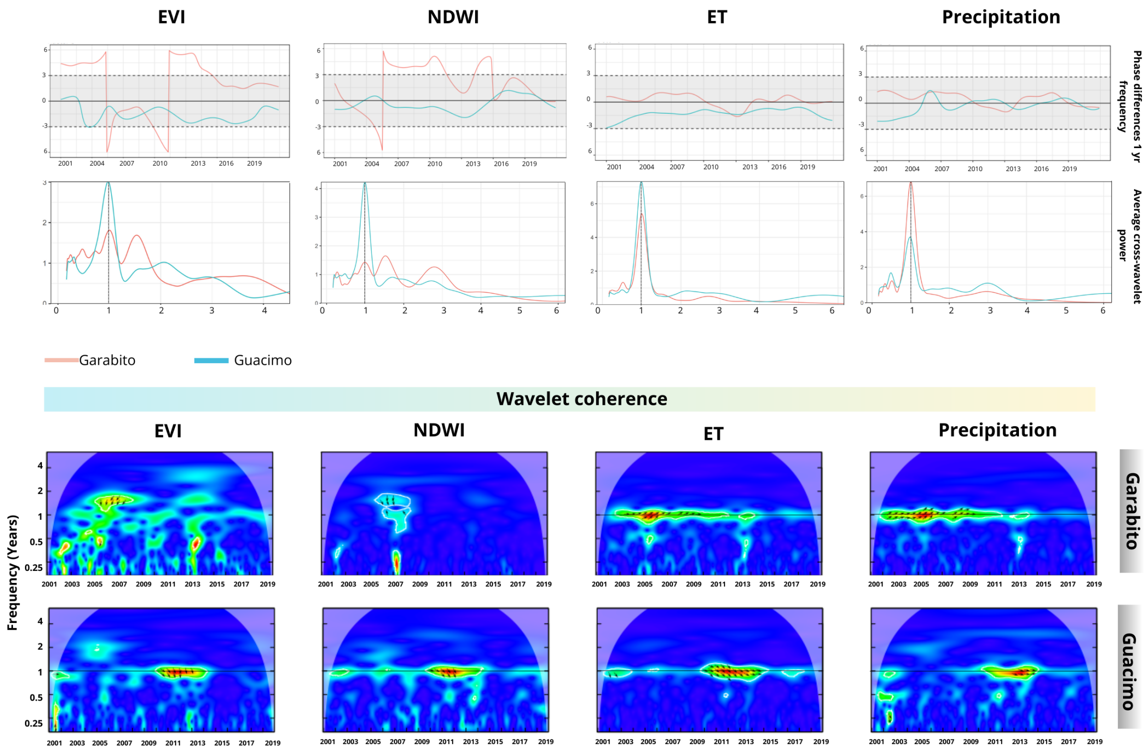

Figure K. Cluster 3.

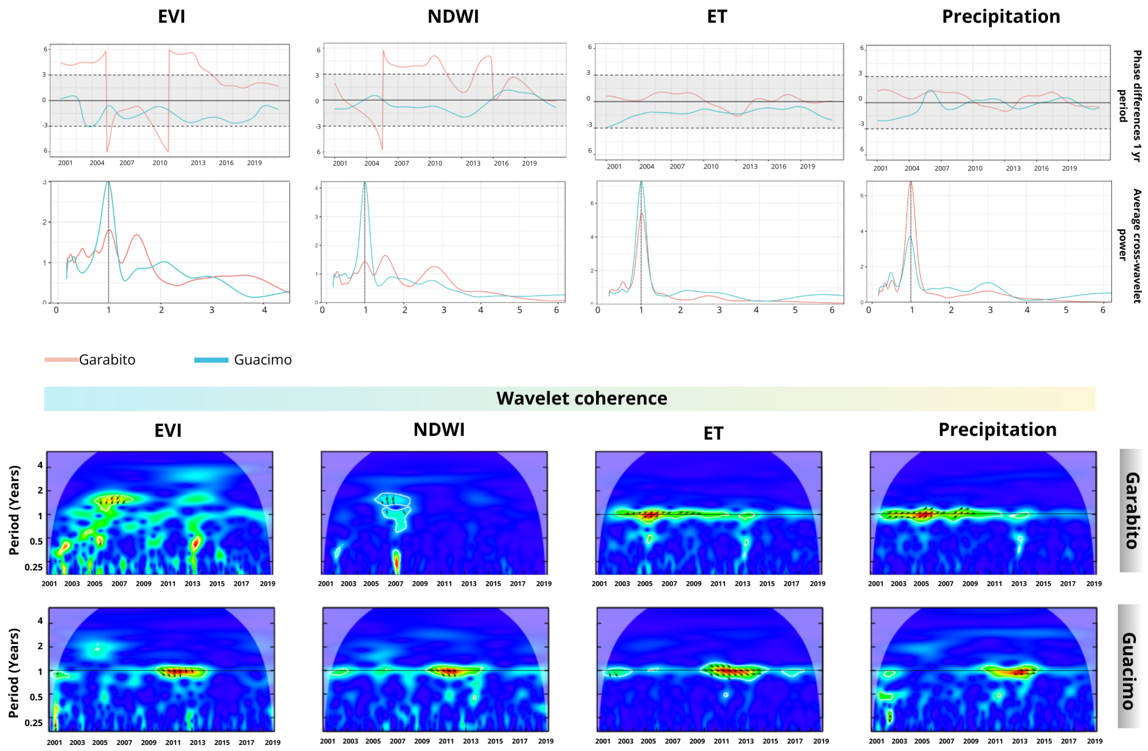

Figure L. Cluster 4.

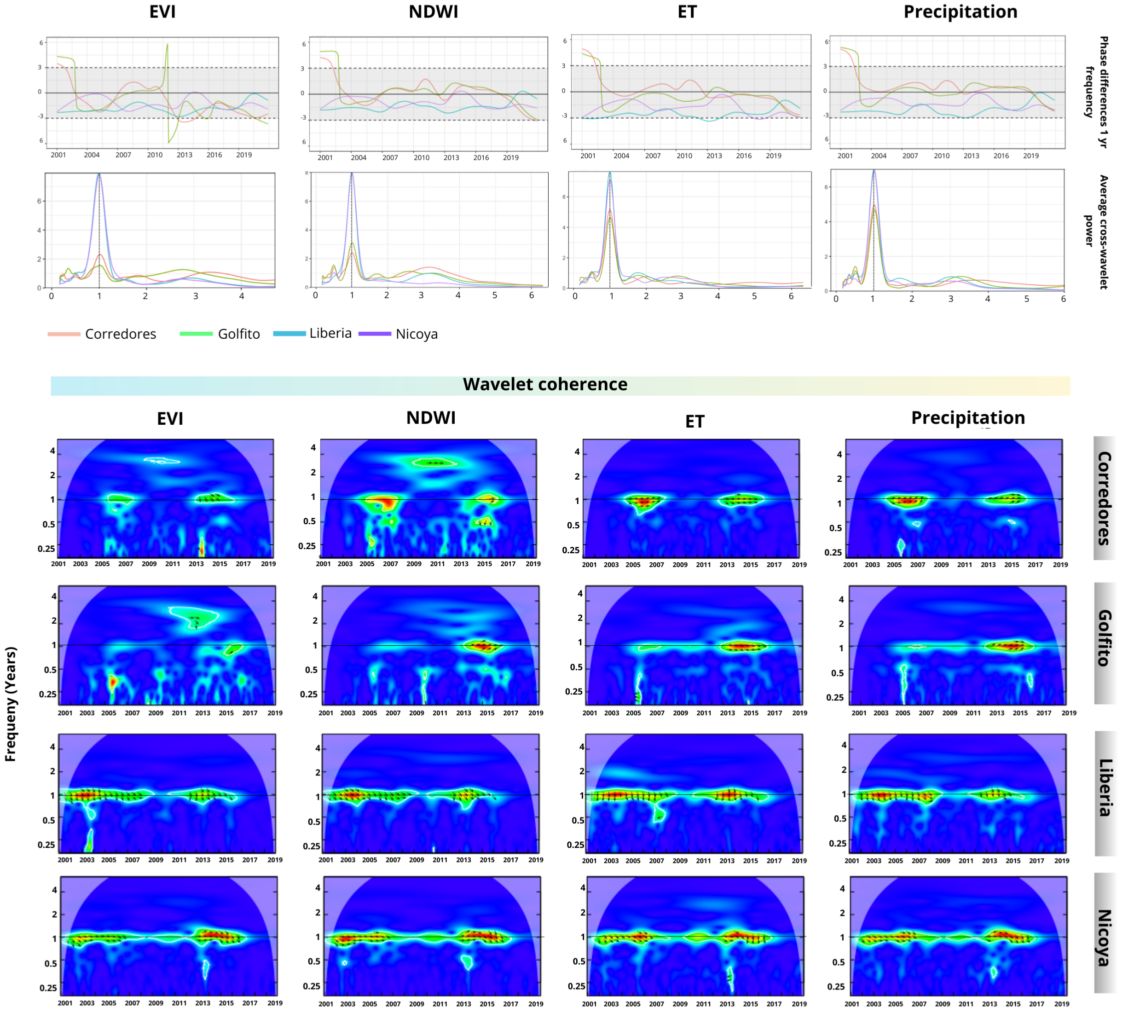

Figure M. Cluster 6.

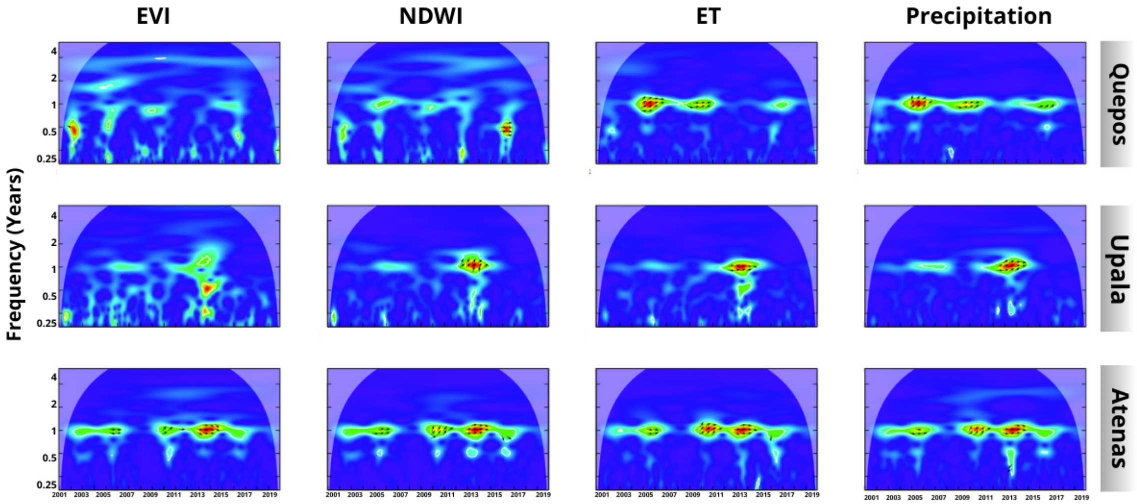

Figure N. Non-clustered cantons

3.3 Other complementary plots

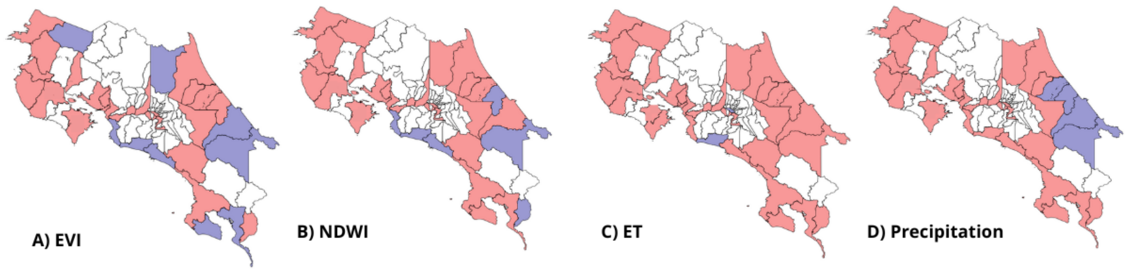

Figure O. Spatial correlation between dengue cases and local environmental variables. The blue-colored cantons indicate regions where no discernible relationship has been identified between the analyzed time series. (Shapefiles are publicly available at (1))

3.3.1 Mean values for all environmental variables from 2001-2019

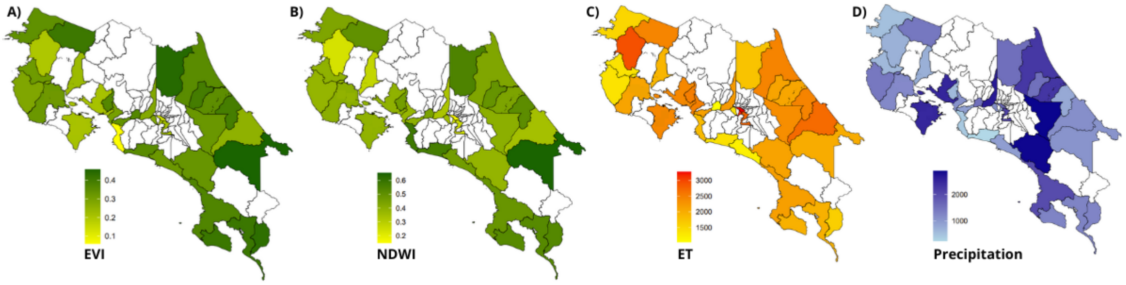

Figure P. Mean values of indices from 2001 to 2019. Figure A) and B) displays the average value of EVI and NDWI for the 32 cantons. A highest values for EVI (darkgreen) indicates a greater extent of healthy and green vegetation in the study area. For NDWI, a highest values (darkgreen) indicates a greater presence of water bodies or moisture content in the study area. Figure C) Represent the average of the ET. When the ET value is higher (red), it indicates a greater rate of water loss, suggesting more active evaporation and transpiration processes. Figure D) represents the average value for precipitation index. (Shapefiles are publicly available at (1))

3.3.2 Clustering only with cases

A preliminary time series clustering analysis, using dynamic time warping similarity and hierarchical clustering, was performed to explore the similarity of dengue cases across the 32 municipalities in Costa Rica. Due to different scales, the logarithmic transformation was the most suitable compared to the squared root. Four groups were identified using dengue behavior, and the groups are matched geographically to some degree (Fig S17). However, it is important to note that our wavelet-based clustering approach accounts for additional factors that the simple clustering cases may not adequately capture. By incorporating climate and local environmental variables, we aim to understand better the underlying relationships and their implications for dengue transmission dynamics.

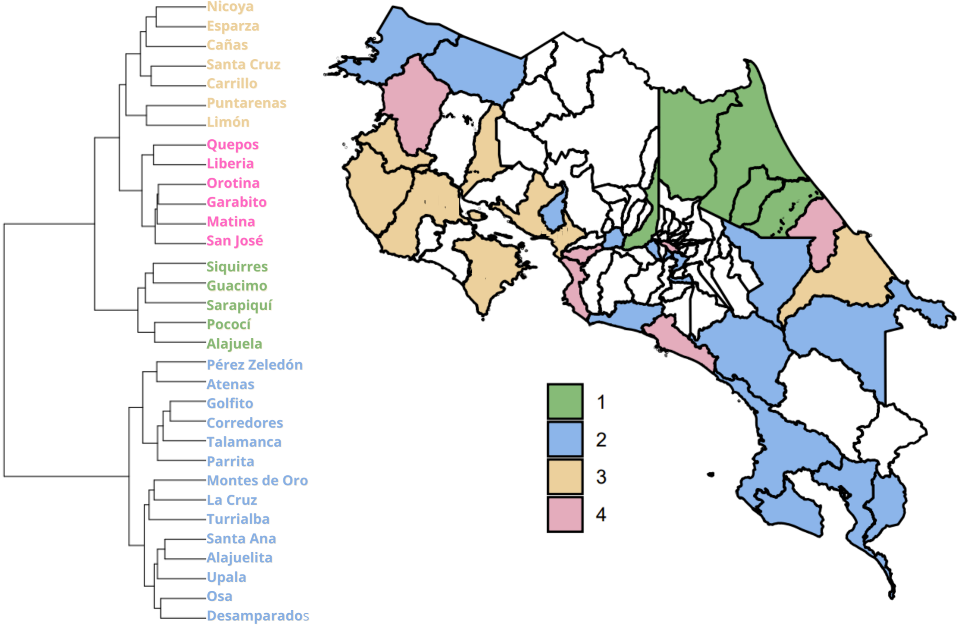

Figure Q. The clustering analysis based on cases has resulted in the identification of distinct clusters. The map illustrates the geographical distribution of cantons within each cluster, as shown in the dendrogram on the left side. The clustering process involved grouping the cantons based on similarities in the number of cases. By examining the dendrogram, we can visualize the hierarchical structure of the clusters. Each branch in the dendrogram represents the merging of two clusters or individual cantons. (Shapefiles are publicly available at (1))

References

1. Global Administrative Areas (GADM). (n.d.). Download country level administrative boundaries. [cited 18 September 2023] Available from:`https://gadm.org/download_country.html`,

2. Cazelles B, Chavez M, McMichael AJ, Hales S. Nonstationary influence of El Niño on the synchronous dengue epidemics in Thailand. PLoS Med. 2005;2(4):e106.

3. Rösch A, Schmidbauer H. WaveletComp 1.1: A guided tour through the R package. 2016. Available from: `http://www.hs-stat.com/projects/WaveletComp/WaveletComp_guided_tour.pdf`.

4. Rösch A, Schmidbauer H, Roesch MA. Package 'WaveletComp'. 2014. The Comprehensive R Archive Network 2014.
